# Creating a Globally Distributed Multinational Dialysis Database - The Apollo*DialDb* Initiative

**DOI:** 10.1016/j.ekir.2025.09.004

**Published:** 2025-09-09

**Authors:** Melanie Wolf, Yue Jiao, Kaitlyn Croft, Carly Hahn Contino, Justin Zimbelman, Kanti Singh, Mitesh Soni, Andrew Dickinson, Jeroen P. Kooman, Dinesh Chatoth, Adrian Guinsburg, Stefano Stuard, Milind Nikam, Michelle Carver, Len Usvyat, Franklin W. Maddux, Sheetal Chaudhuri, John Larkin

**Affiliations:** 1 Renal Research Institute, New York, New York, USA; 2 Fresenius Medical Care Holdings Inc., Waltham, Massachusetts, USA; 3 Privacy Analytics, Ottawa, Ontario, Canada; 4 Faculty of Health Medicine and Life Science, Maastricht University, Maastricht, Netherlands; 5 Fresenius Medical Care, Madrid, Spain; 6 Fresenius Medical Care Deutschland GmbH, Bad Homburg, Hessen, Germany; 7 Fresenius Medical Care, Singapore, Singapore

**Keywords:** end-stage kidney disease, multinational database, outcomes

## Abstract

**Introduction:**

Large amounts of data are captured during dialysis, yet multinational datasets are scarce because of challenges in harmonizing and integrating clinical data, as well as complying with data protection regulations across the world. A global kidney care provider, Fresenius Medical Care, approached this challenge and finalized the creation of an anonymized dialysis database, coined Apollo*DialDb* (Apollo). We report on the approach used for database creation and detail dialysis patient characteristics globally.

**Methods:**

To create this globally distributed multinational database, data from different electronic clinical systems were extracted, covering routinely collected medical information from dialysis clinics worldwide. This data were harmonized, and then anonymized following a reidentification risk assessment conducted by the external company Privacy Analytics, Ontario, Canada. The data was consolidated and is stored in a central cloud environment and will be updated periodically.

**Results:**

Apollo captures data from January 2018 to March 2021 from 40 countries and 543,169 patients worldwide (4.6% in Asia-Pacific [AP], 13.9% in Europe, Middle East, and Africa [EMEA], 7.0% in Latin America [LA], and 74.5% in North America [NA]). It contains demographic data, 35,874,039 laboratory, and 140,016,249 treatment observations as well as frequently recorded medication information, and clinical outcomes (e.g., hospitalization and mortality). Several regional differences can be observed using these data, such as age, treatment modality, and treatment time.

**Conclusion:**

Creating a robust multinational dialysis database offers vast opportunities to conduct real-world research and data analytics, including the development of artificial intelligence models. These activities hold promise of advancing the understanding of kidney disease and dialysis therapies. It can serve as comparative resource for the nephrology community.

## Graphical abstract

**Figure:**
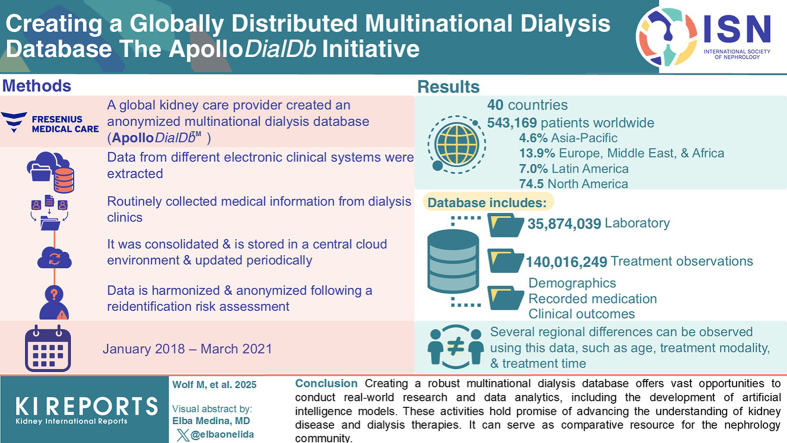

The global incidence and prevalence of chronic kidney disease has significantly increased, and it is rapidly becoming one of the leading causes of death worldwide.^1^ In 2010, 2.62 million individuals underwent dialysis globally, and the demand for this life-saving treatment was expected to double by 2030.^2^ Chronic dialysis can be done in a medical clinic or at home. Hemodialysis (HD) is typically administered 3 times/wk, with each session lasting for about 4 hours, though it can be performed as frequently as daily. In contrast, peritoneal dialysis (PD) is usually performed continuously. Data are captured in a highly granular manner in dialysis care, with multiple measures captured during each dialysis session (e.g., vital signs, machine readings, and medications). Although this vast data can be used for secondary purposes to inform and advance medicine (e.g., continuous quality improvement), it is rarely available in a readily usable form (e.g., anonymized). Anonymized databases are crucial for enabling faster and more accessible data analysis, because they allow researchers to work with large datasets while protecting patients’ privacy. By removing personal identifiers, data can be shared and analyzed more freely, accelerating research, and driving more rapid advancements in health care. A few global anonymized databases already exist in various fields of health care, such as the Global Clinical Platform by the World Health Organization,^3^ the International COVID-19 Open Radiology Database,^4^ or the Cancer Genome Atlas.^5^

In Nephrology, national and international registries have been established to capture a small portion of data generated during dialysis care. Registries are commonly used for public health reporting and oversight and serve as a resource for research and other secondary purposes. A few examples are the European Renal Association registry,^6^ and the United States Renal Data System.^7^ Regional differences exist in registry data with regard to the incidence, prevalence, and causes of end-stage kidney disease, as well as for treatment practices and outcomes.^8^ The explanation for these differences is often speculative for several reasons. First, there is a lack of granularity in registries that do not capture data from most dialysis treatments. Second, inherent heterogeneity exists in national clinical information reporting and data capture methods. Registry data can sometimes be insufficient for distinguishing between the influences of practice patterns versus the underlying pathophysiology of the disease and local conditions and may only offer generalizability within the regional setting.

Comprehensive multinational datasets are scarce and challenging to construct with various data protection regulations in the world. A few examples do exist, such as the MONitoring Dialysis Outcomes (MONDO) initiative^9^ and the Dialysis Outcomes and Practice Patterns Study (DOPPS).^10^ MONDO retrospectively captures observation level data in a joint database representing multiple providers worldwide, with 11 years of longitudinal data from 37 countries across 5 continents.^11^ DOPPS prospectively captures prespecified data from a random sample of clinics at a regular frequency, with > 20 years of longitudinal point-prevalent data from 20 countries across 5 continents.^12^ In an age of big data and artificial intelligence, there is a need for expanding real-world dialysis databases with global representation to drive insights and advance care. A global kidney care provider developed the framework for, and successfully created, a new anonymized globally distributed multinational dialysis database, coined Apollo*DialDb*, that represents dialysis care in 40 countries across 6 continents. This manuscript describes the methods involved and lessons learned in creating a multinational dialysis database with an extensive amount of data from various sources and systems worldwide and provides benchmarks on patient and treatment characteristics.

## Methods

### Data Sources

An integrated kidney care company (Fresenius Medical Care, Bad Homburg, Germany) created a globally distributed multinational anonymized database of patients with end-stage kidney disease called the Apollo*DialDb* (Apollo). The database is governed by a charter defining the oversight, creation, and maintenance of the database, as well as its use for specific projects. To create this database, data from different electronic clinical systems were captured from 4 regional data centers representing operational areas. This included the EuCliD clinical system,^13^^,^^14^ the eCube and Chairside clinical systems^15^ and the affiliated data warehouses. Routinely collected medical information from dialysis patients is available in the systems, along with distinct data captured in specific regions and countries. These data include demographic information, comorbidities, laboratory data, medication as well as information on kidney disease, vascular access, dialysis treatments and clinical outcomes (e.g., hospitalization and mortality).

### Data Identification, Extraction, and Harmonization

Data harmonization is essential to combine data from multiple sources to ensure compatibility and comparability. However, implementing this is challenging, especially when starting with clinical systems with different infrastructure, governance, and requirements in the provision of medical care. Our identification process of potential variables included multiple iterations of thought group sessions and subsequent feasibility investigations with all possible stakeholders worldwide (e.g., clinicians, scientists, statisticians, database engineers, database managers, patient advocates, experts in each given clinical system, as well as legal and privacy experts). This process yielded the backbone of a data dictionary with the planned names and definitions for each variable field, types of data to be captured, and database structure and platform.

**Figure 1:**
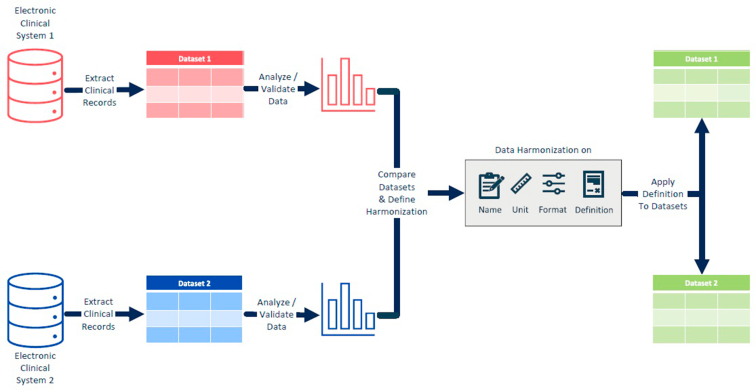
Data harmonization process example for data extractions from 2 electronic clinical systems.

The selected data from the captured medical information were incorporated in the data extraction from the systems (Figure 1). To adhere to data processing regulations, the extraction included data from patients who provided consent for their data to be used for secondary purposes including research. Direct identifiers (variables capable of independently identifying an individual patient, e.g., patient names) were already removed during the data extraction process and replaced by random patient and clinic identifiers. After data extraction, data cleaning revealed areas of missing or structurally problematic data, leading to the exclusion of certain fields and adjustments to the design of the retained fields. To harmonize these retained data, datasets were analyzed to determine differences in data formats, structures, definitions, and semantics. These differences occur from country-to-country even within the same clinical system. To ensure accurate mapping, variables with equivalent meanings must be identified across the different datasets. Based on this analysis, criteria for data harmonization were defined. The names and definitions for all variables were confirmed and revised. For measurements, the same unit was defined for each variable, and values were converted as needed to enable interoperability. All variables were standardized to the same format, be it numeric or text. By applying this definition to the extracted datasets, datasets with the same structure were created, enabling the ability to assess the reidentification risk and combine the datasets after anonymization.

### Legal Aspects and Data Anonymization

Once the data from different datasets are harmonized in a consistent format and structure, an anonymization process is required before the data can be transferred beyond its original environment. The patient’s personal and medical information are confidential and protecting it from unauthorized individuals reduces the risk of misuse and eliminates residual risks after anonymization. Medical information can be used maliciously to commit identity theft, insurance fraud, and other forms of financial crimes.^16^ Health care providers have an ethical duty to prioritize patient safety, including the protection and responsible use of patient information. Patients need to be sure and trust that health care providers protect their sensitive information in order to be willing to consent for further processing of their data. The anonymization process involves the removal and generalization of data that could otherwise be used to identify a natural person. There are numerous laws and privacy regulations with robust data protection measures to ensure that no individual can be identified from the data, whether directly or indirectly, alone or in combination with other data included in the database, such as the Health Insurance Portability and Accountability Act^17^ in the United States and the General Data Protection Regulation^18^^,^^19^ in Europe, that mandate the protection of patient data. In addition, the Brazilian General Personal Data Protection Law (Lei Geral de Proteção de Dados Pessoais) and any applicable local country law need to be considered. Nonadherence can result in penalties and legal consequences for health care providers and organizations.^20^

A reidentification risk determination was performed by Privacy Analytics, an IQVIA company (Ontario, Canada), to create the approach to anonymize data and construct Apollo. The approach satisfied internal best practices, such as ISO/IEC 27559, using a methodology that is published and peer reviewed.^21^, ^22^, ^23^ The Apollo initiative considers the most stringent regulations in any country or region to define a standardized approach for anonymization.

Identifiers (variables that, alone or in combination, may have the potential to identify an individual patient) were reviewed with a team of privacy and data protection experts. The reidentification risk assessment considered the likelihood of individual patients being distinguished using combinations of identifiers within the context of the database.

Given the complexities in sharing personal data with external privacy experts for the reidentification risk assessment, detailed summary data on frequencies, counts, and combinations of identifiers of the datasets were used. In this approach, the summary data on the distributions for identifiers were employed for the reidentification risk assessment and the establishment of the generalization and masking criteria to apply for achieving a risk level lower an established threshold.^24^ These criteria were applied using 1 source of programming that anonymizes the independent datasets and prepares them for migration into a central cloud environment. Once anonymized, the dataset no longer contains information on natural persons and can be used for secondary purposes in accordance with the Declaration of Helsinki and international standards.

### Migration and Storage

**Figure 2:**
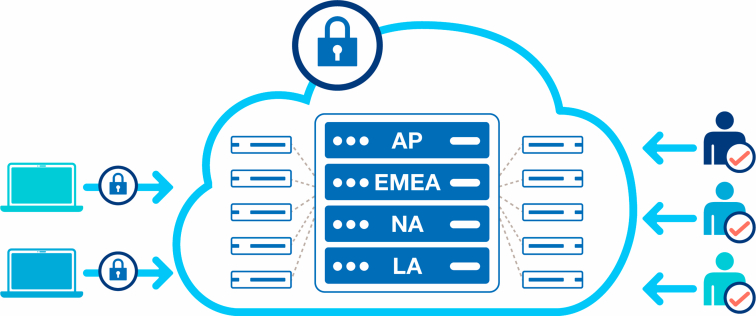
Migration, storage, and access. AP, Asia-Pacific; EMEA, Europe, Middle East, and Africa; LA, Latin Ameria; NA, North America.

After the anonymization process, the datasets were transferred to a secure Amazon Web Services cloud environment server in the USA, hosted by the company using secure methods (Figure 2).

Each table of the anonymized datasets were combined, fields were consolidated and validated to establish the anonymized multinational database Apollo in the cloud. To keep the data protected, only a limited number of authorized people across the company’s regions worldwide have administrative access to this database. A data dictionary was published (Supplementary Material S1), comprising all available variables along with their formats and descriptions, to facilitate a better understanding of the existing dataset. To maximize the database's utility, it is intended to update and expand the data periodically, ensuring the inclusion of the most recent information.

## Results

**Figure 3:**
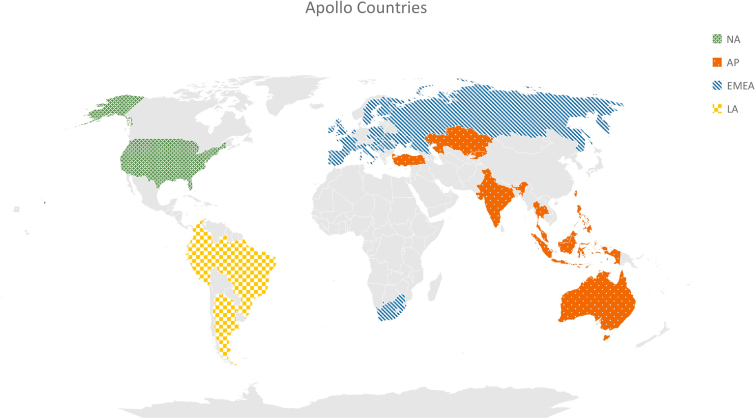
Countries included in the Apollo database. AP, Asia-Pacific; EMEA, Europe, Middle East, and Africa; LA, Latin Ameria; NA, North America.

**Figure 4:**
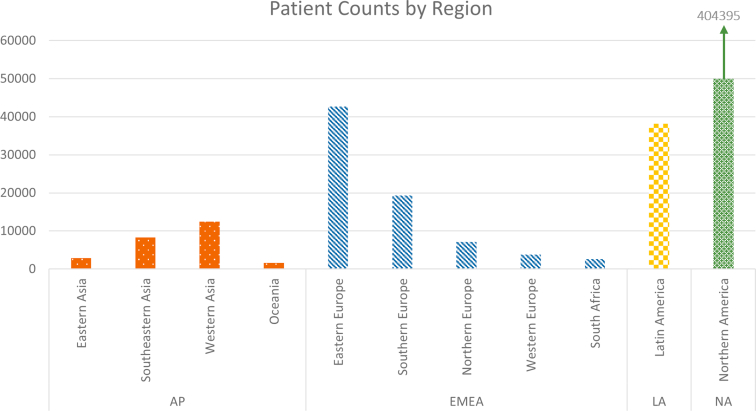
Patient counts by region.

The first version of Apollo contains longitudinal data on > 360 variables from January 2018 to March 2021. The database includes data on 543,169 patients from 40 countries across 6 continents. In Figure 3, we highlight the countries by geographical region. Most of the patients (74.5%) were included in the database from a single country in NA. Of the patients, 4.6% were from 12 countries in AP, 13.9% were from 22 countries in EMEA, and 7.0% were from 5 countries in LA. In Figure 4, we display the distribution of the patients by region. Most of the included patients were from NA. The included countries from LA were represented as 1 region, whereas the countries from EMEA were divided into 5 regions with most patients from Eastern Europe (56.6% of EMEA patients). AP was divided into 4 regions with most patients from Western Asia (49.4% of AP patients) followed by Southeastern Asia (32.9% of AP patients). Several regional differences could be observed. Selected patient characteristics are shown by world region in Table 1.

**Table 1:** Apollo patients characteristics by world region

Parameter	Total	AP	EMEA	LA	NA
Patients, *n*	543,169	25,231	75,396	38,147	404,395
Country, *n*	40	12	22	5	1
Age at dialysis start, yr					
18–44	16.5%	20.5%	16.7%	22.4%	15.6%
45–64	41.2%	46.0%	38.4%	43.4%	41.3%
65–74	24.4%	21.1%	25.3%	21.8%	24.6%
≥ 75	17.9%	12.3%	19.5%	12.4%	18.5%
Female	41.8%	42.2%	40.0%	40.4%	42.3%
Height, cm					
< 150	3.2%	5.2%	4.2%	7.6%	2.4%
150–159	17.8%	27.1%	19.4%	25.8%	16.1%
160–169	33.2%	39.0%	36.6%	39.6%	31.5%
170–179	29.3%	21.6%	30.6%	21.8%	30.2%
180–189	13.5%	3.8%	8.2%	3.2%	16.0%
190–199	1.8%	0.2%	0.7%	0.2%	2.3%
≥ 200	0.10%	0.01%	0.05%	0.02%	0.12%
Ethnicity					
White	52.5%	16.2%	62.4%	31.8%	54.8%
Black	20.6%	0.05%	1.7%	2.3%	27.2%
Asian	2.9%	15.3%	0.2%	0.2%	2.9%

AP, Asia-Pacific; EMEA, Europe, Middle East, and Africa; LA, Latin America; NA, North America.

Data presented as counts (n), proportions (%), or mean values by major world region.

Overall, most patients were aged 45 to 64 years old at dialysis start, yet a higher proportion were younger (18–44 years) in AP and LA; whereas a high proportion of patients were older (65–74 and ≥ 75 years) at dialysis start in EMEA and NA. The proportion of females across regions differed between 40% and 42% overall regions. Most of the patients had heights between 160 and 169 cm in all regions. In EMEA and NA, a high proportion had heights between 170 and 179 cm; whereas a higher proportion of patients in AP and LA had heights between 150 and 159 cm. Most of the patients were of White race, with a higher proportion of Black race being from NA. These differences by region are also visible in treatment and laboratory data. Overall, the database contains information on 35,874,039 laboratory values and 140,016,249 dialysis treatment observations. In Table 2, we present an insight into the distributions for some selected data describing dialysis modalities, treatments, and laboratories.

**Table 2:** Apollo treatment and laboratory patterns by world region

Parameter	Total	AP	EMEA	LA	NA
Patients, *n*	543,169	25,231	75,396	38,147	404,395
Patients by modality^a^, (*n* patients with ≥1 treatment)					
HD	486,384	22,097	51,006	33,426	379,855
HDF	66,088	6447	51,024	8617	*NA*
PD	62,501	*NA*	2409	6617	53,472
HDF patients^a^	66,088	6447	51,024	8617	NA
(*n* patients with ≥1 treatment)					
Pre-dilution	3695	615	3056	24	NA
Post-dilution	61,470	5373	47,775	8322	NA
PD patients^a^	62,501	NA	2409	6617	53,472
(*n* patients with ≥ 1 treatment)					
CAPD	34,785	NA	2185	5637	26,962
CCPD	51,051	NA	374	1677	48,998
TIDAL	283	NA	280	NA	NA
Dialysis treatments, *n*	140,016,249	4,717,899	19,066,011	7,399,095	108,833,244
Hemodialysis treatments					
HD	73.6%	77.1%	36.4%	79.4%	79.6%
HDF	10.5%	22.8%	63.4%	20.5%	NA
Predialysis weight. kg	82.6 ± 23.2	69.5 ± 16.5	76.9 ± 18.5	71.3 ± 16.9	85.4 ± 24.1
Treatment time^b^, min	230.7 ± 33.3	252.7 ± 43.4	249.2 ± 17.2	243.4 ± 11.2	224.1 ± 34.3
Laboratory data, *n*	35,874,039	397,338	2,412,615	668,039	32,396,047
Albumin, g/dl	3.8 ± 0.4	3.9 ± 0.4	3.9 ± 0.5	3.9 ± 0.5	3.8 ± 0.4
Hemoglobin, g/dl	10.8 ± 1.3	11.1 ± 1.8	11.0 ± 1.5	11.1 ± 1.7	10.7 ± 1.3
Ferritin, ng/ml	915.8 (518.3–1285.0)	534.5 (292.1–844.3)	463.4 (256.7–741.4)	642.2 (386.7–981.2)	998.5 (629.0–1331.5)
TSAT, %	32.4 ± 14.2	29.5 ± 14.6	29.3 ± 14.4	30.6 ± 14.8	32.8 ± 14.1
Calcium, mg/dl	8.9 ± 0.7	9.1 ± 0.8	8.8 ± 0.8	8.7 ± 0.7	8.9 ± 0.7
Phosphate, mg/dl	5.3 ± 1.7	4.8 ± 1.4	4.8 ± 1.5	4.6 ± 1.4	5.5 ± 1.7
PTH , ng/l	384.4 (227.2–617.1)	278.4 (151.8–496.6)	303.5 (165.8–519.0)	310.7 (161.4–561.1)	396.6 (237.8–627.9)
Cholesterol, mg/dl	156.0 ± 45.5	165.0 ± 46.0	162.5 ± 45.3	161.9 ± 44.5	147.6 ± 44.5
HDL, mg/dl	41.7 ± 14.2	39.6 ± 14.2	43.6 ± 15.3	38.5 ± 12.3	41.9 ± 13.8
LDL, mg/dl	89.4 ± 37.9	109.7 ± 36.4	92.3 ± 37.6	94.5 ± 38.5	73.7 ± 32.2
WBC, × 10ˆ3/μl	6.9 ± 3.4	6.5 ± 3.8	6.8 ± 5.4	7.0 ± 2.6	6.9 ± 2.9

AP, Asia-Pacific; CAPD, continuous ambulatory peritoneal dialysis; CCPD, continuous cycling peritoneal dialysis; EMEA, Europe, Middle East, and Africa; HD, hemodialysis; HDF, hemodiafiltration; HDL, high density lipoprotein; LA, Latin America; LDL, low density lipoprotein; NA, North America; PD, peritoneal dialysis; PTH, parathyroid hormone; TSAT, transferrin saturation; WBC, white blood cell.

Data presented as counts (n), proportions (%), mean ± SD, or median [q1–q3] values by major world region.

a Patients are counted for each modality they have received at least once. Because patients can switch between modalities (HD, HDF, and PD), some are counted multiple times; once for each treatment they have undergone. Similarly, the numbers of different types of PD treatments (CAPD, CCPD, Tidal) reflect patients being counted once for each type of PD treatment they have received.

b HD and HDF treatments are included for the average of treatment time.

The most common modality in EMEA was hemodiafiltration with postdilution, and HD was most common in the other regions. PD was more frequently used by patients in LA (17%) and in NA (13%) than in other regions. Continuous cycling peritoneal dialysis was mainly used in NA, whereas continuous ambulatory peritoneal dialysis was the common PD treatment in LA and EMEA. The mean treatment time was lower in NA; on average, the treatment time was 19 to 28 minutes shorter than in other regions. There was a difference in mean predialysis bodyweight > 15 kg between regions; the highest weights were observed in EMEA and NA in contrast to the AP and LA regions. Mean albumin levels were slightly < 4.0 g/dl and consistent in all regions; the mean hemoglobin levels were same. Ferritin and transferrin saturation levels were the lowest in EMEA and highest in NA. Mineral bone disorder laboratories showed mean values within the target range. Cholesterol and low-density lipoprotein levels were the lowest in NA compared with other regions. White blood cell counts were relatively unremarkable and consistent across regions.

## Discussion

The Apollo database is a globally distributed anonymized multinational dialysis database. With the data from > 500,000 patients in 40 countries across 6 continents, it offers the opportunity to enable and streamline worldwide data analytics to advance the field of medicine. National registries made from point-prevalent data cannot fully address the same research questions as a multinational database that encompasses all observations and measures captured in care from a broad dialysis patient population. It is essential to have a multinational database to allow worldwide analysis of a diverse population and to identify regional patterns. As indicated in Table 2, there are noticeable differences in practice patterns across regions, such as the shorter average treatment time in NA, which is 19 to 28 minutes less than in other regions.

In Table 3, we compare patient characteristics of the Apollo database with the existing multinational databases MONDO and DOPPS. An upcoming new version of MONDO offers data of 20 years from 2000 to 2019 and includes almost 290,000 patients from 41 countries.^25^ DOPPS started in 1996 to collect data on dialysis patients in the USA and has extended this to > 20 countries. The annual report from 2012 delivers insights into data from 1997 to 2011 for patients on HD.^26^ Some additional data are publicly available through the DOPPS Practice Monitor that offers the opportunity to directly access data and descriptive analysis from patients treated between 2010 and 2021. Most recent data, like the Apollo timeframe ending in 2021, are available in the Practice Monitor for patients from the USA (data from 2010–2021) and from Japan (data from 2013–2021).^27^ The Peritoneal Dialysis Outcomes and Practice Patterns Study, a specialized version of DOPPS, aimed at enhancing the understanding of best practices for patients on PD globally and provides valuable data on PD patients. Nine countries provided data so far on 11,688 consented patients on PD.^28^ The DOPPS Practice Monitor provides publicly PD data only for the USA but not for other countries.^28^

**Table 3:** Patient characteristics across different multinational databases

Characteristics	Apollo	MONDO		DOPPS						PDOPPS^a^
Timeframe	2018–2021	2000–2019		1997–2011^b^	2010–2021^c^	2012–2018^c^	2012–2016^c^	2013–2021^c^	2016–2018^c^	2019–2022
Patients *n*	543,169	289,715		29,013	12,358	2845	1754	4665	5308	3762
Countries *n*	40	41		12	1 (USA)	1 (Canada)	1 (Germany)	1 (Japan)	6 (GCC)	1 (USA)
Age, yr										
18–44	16.5%	N/A	18–34:	3.7%	1.0%	3.9%	3.0%	4.2%	10.9%	4.9%
45–64	41.2%	N/A	35–54:	20.0%	24.7%	18.9%	17.2%	21.1%	30.1%	27.2%
65–74	24.4%	N/A	55–74:	47.7%	51.2%	45.9%	43.6%	43.7%	47.3%	49.7%
≥ 75	17.9%	N/A	≥ 75:	28.6%	23.1%	31.2%	36.1%	30.9%	11.6%	18.2%
Female	41.8%	41.2%		40.2%	42.6%	40.6%	37.7%	32.4%	44.8%	43.5%
Ethnicity										
White	52.5%	N/A		60.4%	*N/A*	68.3%	97.7%	*N/A*	*N/A*	*N/A*
Black	20.6%	N/A		10.8%	30.9%	7.6%	0.7%	*N/A*	*N/A*	19.4%
Asian	2.9%	N/A		22.7%	*N/A*	5.4%	0.6%	*N/A*	*N/A*	*N/A*
Modality										
HD	89.5%	83.6%		100%	100%	100%	100%	100%	100%	*N/A*
HDF	12.2%	37.4%								
PD	11.5%	11.3%		N/A	N/A	N/A	N/A	N/A	N/A	100%

DOPPS, Dialysis Outcomes and Practice Patterns Study; GCC, Gulf Cooperation Council (Bahrain, Kuwait, Oman, Qatar, Saudi Arabia, and United Arab Emirates); HD, hemodialysis; HDF, hemodiafiltration; PD, peritoneal dialysis; PDOPPS: Peritoneal Dialysis Outcomes and Practice Patterns Study.

a Data from DOPPS Practice Monitor for PD patients (characteristics presented are from last available year).

b DOPPS: 2012 Annual Report, Data from 2011 for HD, European Countries (France, Germany, Italy, Spain, UK) started in 1998, Japan in 1999, Australia, Belgium, Canada, New Zealand, and Canada started in 2002.

c Data from DOPPS Practice Monitor for patients on HD (characteristics presented are from last available year).

In Table 3, we show differences between these databases. Compared with Apollo and DOPPS, MONDO offers data from pediatric patients.^29^ The age grouping in the Apollo and MONDO database is consistent with the United States Renal Data System. DOPPS uses a different age grouping, making it challenging to compare patient distributions. In most regions, the randomly selected patients included in DOPPS tend to be older than in Apollo (for HD and PD). Most of the patients are aged between 55 and 74 years; in some countries, a high proportion is aged ≥ 75 years. Female patients account for approximately 40% of all patients in all databases. However, this percentage is slightly lower in Germany and Japan, and slightly higher in the Gulf countries according to the DOPPS database. Apollo has a high number of missing values for race; whereas in DOPPS, there is good documentation for the countries included in the annual report for 2012. In Apollo and MONDO, a differentiation is made between HD and hemodiafiltration, which makes comparisons possible. In addition, patients on PD are included in the databases.

To fully represent the broader dialysis patient population, it is important to be able to address the needs and outcomes of diverse patient groups, and of those who might be underrepresented. A broader dialysis patient population provides a comprehensive understanding of dialysis practices and outcomes. Compared with DOPPS and MONDO, Apollo emphasizes not only epidemiological insights but also operational insights. Apollo includes data from more patients and more data elements than existing databases. Apollo is designed to be routinely updated and expanded, capturing information on every observation for each variable that is captured in real-world care, versus period point-prevalent information. With its frequent updates, expanding information on fields and time periods, Apollo supports continuous learning at the point of care. It enables the development of artificial intelligence–based prediction tools and offers a broader perspective on a large, multinational patient population. Apollo provides a valuable, real-world complement to broader, multisource initiatives such as DOPPS and MONDO. It offers a unique opportunity to examine end-stage renal disease care delivery and outcomes at scale, under standardized conditions, and with direct applicability to clinical decision support, and changes over time.

Compared with the other presented multinational databases, the Apollo database has several strengths. To the best of our knowledge, it is the first multinational database that covers such a broad dialysis patient population with > 500,000 patients from 40 countries across 6 continents, > 140 million treatments, and frequently recorded laboratory and outcome data from regions worldwide. The data have been harmonized and anonymized to comply with all data protection regulations. By rendering personal data on a natural person anonymously, it provides a dataset that complies with international standards and principles. The data are readily available and can be used immediately for analysis to answer specific research questions without requiring extensive data management beforehand. For example, questions such as “impact of fluid overload changes within the first 6 months of dialysis on hospitalization during a 2-year follow-up”^30^ or “profiles of home medication use in patients on dialysis globally”^31^ can be explored. These represent only 2 examples and the dataset enables a wide array of future research, including investigations into patient outcomes, modality use, and predictive modeling. This database enables comprehensive analysis to gain a better understanding of the population of globally distributed patients on dialysis. Comparing outcomes and practices worldwide could lead to new insights and quality improvement initiatives, as well as better patient outcomes. Storing the database in the cloud enables authorized consumers anywhere in the world to access the data for analysis easily. In addition, it gives the opportunity to accommodate the vast amount of data that Apollo covers and provides flexibility for the updates of the database with a longer timeframe and incorporating even more data. Maintenance reidentification risk determinations will be performed to ensure that the anonymization criteria are up-to-date and compliant with the latest guidelines and data privacy regulations and afford opportunities to expand data elements captured.

Apollo has some limitations. Although Apollo offers data on a diverse and large number of patients, the geographic coverage of the dataset is limited because it reflects only regions and countries where the company operates. It may not provide a comprehensive global perspective and may introduce potential selection bias. By the fact that the data represent patients treated exclusively within this single private provider network, the findings may be influenced by operational standards and may not fully represent practices in other health care systems or public-sector settings as well as national-level variations in policy or funding. To achieve anonymization, some data (e.g., age, height, first data of dialysis) was generalized. Thus, analysis of trends within periods < 1 year (e.g., seasonal patterns) is not possible with the current version of the database. Moreover, pediatric patients were excluded to achieve anonymization and maintain as much granular clinical information as possible in the dataset. As a result, the findings will not be generalizable to the pediatric end-stage renal disease population. In addition, although a high number of patients have been included, patients on PD are not represented in every region. An additional limitation is the presence of missing values for certain variables, notably race or ethnicity. Because this dataset is intended for open secondary analysis, we did not apply imputation or exclude entries with missing data, leaving such decisions to end users based on their specific needs and analytical frameworks. The missingness of data may need to be carefully evaluated in future studies using this resource.

It is planned to update Apollo periodically (e.g., annually) to keep the database current. Additional variables can be added during maintenance reidentification risk assessments if available and needed. The timeframe can be adjusted to provide access to a larger dataset, including even more patients and additional data. However, with more data comes the potential for increased risk because of new identifiers and longer timeframes, which may require changes in the anonymization criteria.

## Conclusion

In conclusion, this globally distributed multinational Apollo*DialDb* database is unique and offers vast opportunities to conduct real-world research and various data analytics activities, including development of artificial intelligence models. These activities hold promise of advancing the understanding of kidney disease and dialysis therapies. The data from this multinational database serve as comparative resource for the nephrology community and may complement broader registry-based initiatives. These findings may contribute to evidence-based decision-making in nephrology. Future database updates will include longer timeframes with more data (e.g. intradialytic measures, remote monitoring information, and expanded patient reported outcomes), as well as information on dialysis in acute kidney injury, which will be useful to answer even more research questions.

## Disclosure

MW, KC, and SS report being employees of Fresenius Medical Care Deutschland GmbH. CHC, KS, MS, DC, and MC report being employees of Fresenius Medical Care Holdings Inc. YJ, JZ, SC, LU, and JWL report being an employee of the Renal Research Institute LLC, a wholly owned subsidiary of Fresenius Medical Care Holdings Inc. AG reports being an employee of Fresenius Medical Care Spain. MN reports being an employee of Fresenius Medical Care Asia Pacific. FWM reports being an employee of Fresenius Medical Care AG. SC, DC, LU, FWM, and JWL report having share options or ownership in Fresenius Medical Care. SC, DC, LU, FWM, and JWL report being inventors on patent(s) in the field of dialysis. JWL reports receipt of honorarium from The Lancet; being on the Editorial Board of Frontiers in Physiology and Frontiers in Medicine, Nephrology; being chairperson for a MONitoring Dialysis Outcomes (MONDO) Initiative study group; and serving on the MONDO Steering Committee. LU reports being an advisory board member for Privacy Analytics Inc. FWM reports directorships in Fresenius Medical Care Management Board and Vifor Fresenius Medical Care Renal Pharma. AD reports being employee of Privacy Analytics. FWM, LU, and MC report being members of the Apollo Steering Committee.

## Supplementary Material

Supplementary File (PDF) Data dictionary.

## References

[1] Staplin N. (2024). A global view on kidney care. Nephrol Dial Transplant.

[2] Liyanage T., Ninomiya T., Jha V. (2015). Worldwide access to treatment for end-stage kidney disease: a systematic review. Lancet.

[3] The WHO global clinical platform. World Health Organizatio. https://www.who.int/tools/global-clinical-platform.

[4] Tsai E.B., Simpson S., Lungren M.P. (2021). The RSNA International COVID-19 Open Radiology Database (RICORD). Radiology.

[5] Center for Cancer Genomics. National Cancer Institute The Cancer Genome Atlas Program (TCGA). National Institutes of Health. https://www.cancer.gov/ccg/research/genome-sequencing/tcga.

[6] Boerstra B.A., Boenink R., Astley M.E. (2024). The ERA Registry Annual Report 2021: a summary. Clin Kidney J.

[7] Johansen K.L., Gilbertson D.T., Li S. (2024). US renal data system 2023 annual data report: epidemiology of kidney disease in the United States. Am J Kidney Dis.

[8] Stel V.S., Boenink R., Astley M.E. (2024). A comparison of the epidemiology of kidney replacement therapy between Europe and the United States: 2021 data of the ERA Registry and the USRDS. Nephrol Dial Transplant.

[9] Usvyat L.A., Haviv Y.S., Etter M. (2013). The monitoring dialysis outcomes (MONDO) initiative. Blood Purif.

[10] Pisoni R.L., Gillespie B.W., Dickinson D.M., Chen K., Kutner M.H., Wolfe R.A. (2004). The dialysis outcomes and practice patterns study (DOPPS): design, data elements, and methodology. Am J Kidney Dis.

[11] Chaudhuri S., Larkin J., Guedes M. (2023). Predicting mortality risk in dialysis: assessment of risk factors using traditional and advanced modeling techniques within the Monitoring Dialysis Outcomes initiative. Hemodial Int.

[12] Dialysis Outcomes and Practice Patterns Study (DOPPS) Studies within the DOPPS program. https://www.dopps.org/OurStudies.aspx.

[13] Marcelli D., Kirchgessner J., Amato C. (2001). EuCliD (European Clinical Database): a database comparing different realities. J Nephrol.

[14] Barbieri C., Neri L., Stuard S., Mari F., Martín-Guerrero J.D. (2023). From electronic health records to clinical management systems: how the digital transformation can support healthcare services. Clin Kidney J.

[15] Kalathil R. (2009). eCube combines clinical and billing applications. Nephrol News Issues.

[16] Koppel R., Kuziemsky C. (2019). Healthcare data are remarkably vulnerable to hacking: connected healthcare delivery increases the risks. Stud Health Technol Inform.

[17] Edemekong P.F., Annamaraju P., Afzal M., Haydel M.J. (2024). StatPearls.

[18] Mondschein C.F., Monda C., Kubben P., Dumontier M., Dekker A. (2019). Fundamentals of Clinical Data Science.

[19] (2016). Regulation (EU) 2016/679 of The European Parliament and of the Council of 27 April 2016 on th eprotection of natural persons with regard to the processing of personal data and on the free movement of such data, and repealing Directive 95/46/EC (General Data Protecion Regulation).

[20] Alder S. (Posted December 20, 2023). The cost of non-compliance with HIPAA. The HIPAA Journal.

[21] Arbuckle L., Mian M.O. (2020). Engineering risk-based anonymisation solutions for complex data environments. J Data Prot Privacy.

[22] Arbuckle L., El Emam K. (April 2020).

[23] Arbuckle L., Ritchie F. (2019). The five safes of risk-based anonymization. IEEE Secur Priv.

[24] Elamir E.A.H., Domingo-Ferrer J., Torra V. (PSD 2004). Privacy in Statistical Databases.

[25] Rigodon V, Guedes M, Tiv S, et al. Characteristics of global dialysis data from multiple providers in the new MONitoring Dialysis Outcomes (MONDO) Dataset. *JASN*. 34(11S):175-176. https://doi.org/10.1681/ASN.20233411S1175c

[26] DOPPS Annual Report 2012. https://www.dopps.org/annualreport/.

[27] DOPPS Practice Monitor. https://www.dopps.org/DPM-HD/Default.aspx.

[28] The Peritoneal Dialysis Outcomes and Practice Patterns Study. https://www.dopps.org/OurStudies/PeritonealDialysisPDOPPS.aspx.

[29] von Gersdorff G.D., Usvyat L., Marcelli D. (2013). Monitoring dialysis outcomes across the world--the MONDO Global Database Consortium. Blood Purif.

[30] Wolf M., Ficociello L., Zhou M. (2024). Changes in fluid overload during the first 6 months of dialysis among more than 13,000 patients. J Am Soc Nephrol.

[31] Feuersenger A., Roth L., Wolf M. (2024). Profiles of home medication use in patients on dialysis globally. J Am Soc Nephrol.

