# Supplementary material for: Creating a Globally Distributed Multinational Dialysis Database - The ApolloDialDb Initiative
Source: Kidney Int Rep. 2025 Sep 9;10(11):3855–64. doi: 10.1016/j.ekir.2025.09.004 (PMC12640056; doi:10.1016/j.ekir.2025.09.004)
Supplement: Supplementary File (PDF) — Data dictionary. [file mmc1.pdf]

List of Tables

Table Name	Notes
Demographics_DB	
BCM_DB	Not available in FMCNA
Comorbids	FME in AP, LatAm, and EMEA do not have ICD 9 data, but do have ICD 10 data.
Drug_Home	
Drug_Incenter	
ECHO_DB	Not available in FMCNA
Event_DB	
Lab_DB	Note: We are keeping all valid data on labs, which can include more than one of the same lab values on one specific calendar date. This can cause two rows for one patient on the same date, with the second row only including data on the lab and no other information. FMCNA and FME AP do not have data on blood type.
TXT_DB	Note: For cohort definition, require >=1 hemodialysis treatment or >=1 PD prescription/treatment.<br>Note: FME in EMEA and AP do not have data on txt location (home or in-center), but do have modality type.<br>Note: We are keeping all valid data on treatments, which can include more than one of the same specific calendar date. This can cause two rows for one patient on the same date.
QOL	Not available in FME EMEA, AP, and LatAm.

Demographics_DB

Number	Variable	Variable Type	Description	Unit	Cleaning rules Min valid value	Cleaning rules Max valid value
1	GFME_ID	Num	**Primary key**: Randomly assigned patient identification number.			
2	COUNTRY	Char	Country where patient received dialysis.			
3	DEMO_ESRD_CAUSE_ICD10TEXT	Char	ICD 10 code for the first / primary cause of kidney failure (i.e. end stage kidney/renal failure).	ICD 10 code		
4	DEMO_HEIGHT	Char	Height of the patient as recorded in the medical record. Height is categorized into 10 cm groups with a low and high cutoff (<150, 150-159, 160-169, 170-179, 180-189, 190-199, >200).	Height groups in cm	80	250
5	PROVIDER	Char	Name of the dialysis provider contributing data for the Apollo DB.			
6	DEMO_FDD	Num	The first date of dialysis (FDD) for kidney failure (i.e. end stage kidney failure), which will be provided as the year of FDD.	Year	1985	2021
7	DEMO_AGE_FDD	Char	Age of each distinct patient at the first date of dialysis (FDD) for kidney failure (i.e. end stage kidney failure).	Age groups in years	18	122
8	DEMO_MALE	Num	Biological sex of the patient. This field defines if the sex is male = "1" or female = "0". Data on other sex categories will not be captured and will be considered missing.			
9	DEMO_RACE	Char	Race of the patient. Race is categorized into one of the following specific groups (Asian, Black, White or Other) if applicable.			
10	REGION	Char	World region where the patient was treated.			

BCM_DB

Number	Variable	Variable Type	Description	Unit	Cleaning rules<br>Min valid value	Cleaning rules<br>Max valid value
1	GFME_ID	Num	**Primary key**: Randomly assigned patient identification number.			
2	DAYS_FROM_FDD	Num	Days between the first date of dialysis (FDD) for kidney failure (i.e. end stage kidney failure) and the date that the body composition monitor/analyzer (BCM) reading was performed on the patient. There can be more than one readings on the same date.	Days	0	
3	BCM_HEIGHT	Num	Height used for body composition monitor (BCM) reading. Height is categorized into 10 cm groups with a low and high cutoff (<150, 150-159, 160-169, 170-179, 180-189, 190-199, >200).	Height groups in cm	80	250
4	BCM_ATM	Num	Adipose Tissue Mass (ATM) measurement from body composition monitor (BCM) reading.	Kg	0	300
5	BCM_BCM	Num	Body Cell Mass (BCM) measurement from body composition monitor (BCM) reading.	Kg	0	300
6	BCM_CAPACITY	Num	Cell membrane capacitance measurement from body composition monitor (BCM) reading.	nF		
7	BCM_ECW	Num	Extracellular fluid/water (ECW) measurement from body composition monitor (BCM) reading.	l	0	300
8	BCM_ERROR	Num	Error measurement from body composition monitor (BCM) reading.			
9	BCM_EXTRACELLULAR_RESISTANCE	Num	Extracellular resistance measurement from body composition monitor (BCM) reading.	Ohm		
10	BCM_FTM	Num	Fat tissue mass measurement from body composition monitor (BCM) reading.	Kg	0	300
11	BCM_ICW	Num	Intracellular fluid/water (ICW) measurement from body composition monitor (BCM) reading.	l	0	300
12	BCM_INTRACELLULAR_RESISTANCE	Num	Intracellular resistance measurement from body composition monitor (BCM) reading.	Ohm		
13	BCM_LTM	Num	Lean Tissue Mass (LTM) measurement from body composition monitor (BCM) reading.	Kg	0	300
14	BCM_NORMOHYDRATION_WEIGHT	Num	Normohydration weight measurement from body composition monitor (BCM) reading.	Kg	10	300
15	BCM_QUALITY		Quality measurement from body composition monitor (BCM) reading.	%	0	100
16	BCM_TBW	Num	Total body fluid/water (TBW) measurement from body composition monitor (BCM) reading.	l	0	300
17	BCM_PRE_WEIGHT	Num	Pre-dialysis weight used for body composition monitor (BCM) reading.	Kg	20	300
18	BCM_DRY_WEIGHT	Num	Estimated dry weight from body composition monitor (BCM) reading.	Kg	20	300
19	BCM_TIME_DELAY	Num	Cole parameter phase/time delay (T_d) measurement from body composition monitor (BCM) reading.	ns		
20	BCM_ALPHA	Num	Cole parameter Alpha measurement from body composition monitor (BCM) reading. Cole parameter Alpha is caused by distribution of time constants in biological tissue.		0	1

Comorbids

Number	Variable	Variable Type	Description	Unit	Cleaning rules Min valid value	Cleaning rules Max valid value
1	GFME_ID	Num	**Primary key**: Randomly assigned patient identification number.			
2	COMORBID_START_DAYS_FROM_FDD	Num	Days between the first date of dialysis (FDD) for kidney failure (i.e. end stage kidney failure) and the date that the comorbid condition had onset/started. This can be a negative number if the comorbidity started before the FDD date.	Days		
3	COMORBID_END_DAYS_FROM_FDD	Num	Days between the first date of dialysis (FDD) for kidney failure (i.e. end stage kidney failure) and the date that the comorbid condition ended/stopped. Data from comorbidities that ended before the FDD date is not captured.	Days	0	
4	COMORBID_ICD10TEXT	Char	ICD 10 code for each comorbidity if applicable.	ICD 10 code		
5	COMORBID_ICD9TEXT	Char	ICD 9 code for each comorbidity if applicable.	ICD 9 code		

Drug_Home

Number	Variable	Variable Type	Description	Unit	Cleaning rules Min valid value	Cleaning rules Max valid value
1	GFME_ID	Num	**Primary key**: Randomly assigned patient identification number.			
2	DAYS_FROM_FDD_START	Num	Days between the first date of dialysis (FDD) for kidney failure (i.e. end stage kidney failure) and the start date for the use or prescription of the medication. This can be a negative number if the medication was started before the FDD date.	Days		
3	DAYS_FROM_FDD_END	Num	Days between the first date of dialysis (FDD) for kidney failure (i.e. end stage kidney failure) and the end date for the use or prescription of the medication (i.e. when the medication was discontinued). Data from medications that were discontinued before the FDD date is not captured.	Days	0	
4	DRUG_DOSE	Char	Drug dosage of the medication per administration. This is the amount/dose of a medication prescribed to be taken/administered at a given frequency noted in the field DRUG_FREQUENCY.			
5	DRUG_DOSE_MU	Char	Measurement unit of the drug dose for the prescribed medication. This can be expressed as a weight, percent, volume, number, or quantity and is anticipated to accompany data in the DRUG_DOSE field.			
6	DRUG_DOSE_QTY	Num	Quantity/number of drug doses for the prescribed medication per administration. This is the prescribed quantity of the medication to be taken/administered each time, such as the number of tablets, applications, or inhalations per administration. The data on quantity is anticipated to accompany data in the DRUG_DOSE field.			
7	DRUG_FREQUENCY	Char	Frequency of the drug dose to be taken/administered per duration in time. This details how often the drug dose is prescribed to be taken/administered, such as how many times per day, week, or month. The data on frequency describes the data in the DRUG_DOSE field over time.			
8	DRUG_ROUTE	Char	Route of the medication. This is the route that was used to administer the medication to the patient as recorded in the medical record.			
9	DRUG_NAME	Char	Drug name in text format. This is the name of the prescribed medication.			
10	DRUG_ATC_CODE	Char	The Anatomical Therapeutic Chemical (ATC) code for the medication. ATC code is the unique classification system defined by the World Health Organization for a medication as related to the organ or system it works on and its mechanism of action.	ATC code		
11	DRUG_NDC_CODE	Char	The National Drug Code (NDC) for the medication. The NDC is a unique classification system defined by the United States Food and Drug Administration (FDA) for medications that are regulated by the FDA. This may not be applicable to providers outside the United States.	NDC code		

Drug_Incenter

Number	Variable	Variable Type	Description	Unit	Cleaning rules Min valid value	Cleaning rules Max valid value
1	GFME_ID	Num	**Primary key**: Randomly assigned patient identification number.			
2	DAYS_FROM_FDD	Num	Days between the first date of dialysis (FDD) for kidney failure (i.e. end stage kidney failure) and the date the in-center medication was taken/administered at the dialysis clinic.	Days	0	
3	DRUG_DOSE	Num	Drug dose administered to the patient at a dialysis treatment. This is the amount/dose of a medication taken/administered to the patient at each dialysis treatment.			
4	DRUG_DOSE_MU	Char	Measurement unit of the drug dose for the administered medication. This can be expressed as a weight, percent, volume, number, or quantity and is anticipated to accompany data in the DRUG_DOSE field.			
5	DRUG_ROUTE	Char	Route of the medication. This is the route that was used to administer the medication to the patient during the dialysis treatment.			
6	DRUG_NAME	Char	Drug name in text format. This is the name of the administered medication.			
7	DRUG_ATC_CODE	Char	The Anatomical Therapeutic Chemical (ATC) code for the medication. ATC code is the unique classification system defined by the World Health Organization for a medication as related to the organ or system it works on and its mechanism of action. This data is only required for providers who are capturing this in the medical records during the standard care of dialysis patients.	ATC code		
8	DRUG_INDICATION	Char	ICD-10 code for why the drug is indicated to be used and administered at the clinic. The indication will be related to a comorbidity or medical condition, including dialysis dependent kidney failure (e.g. end stage kidney disease).	ICD 10 code		

ECHO_DB

Number	Variable	Variable Type	Description	Unit	Cleaning rules Min valid value	Cleaning rules Max valid value
1	GFME_ID	Num	**Primary key**: Randomly assigned patient identification number.			
2	DAYS_FROM_FDD	Num	Days between the first date of dialysis (FDD) for kidney failure (i.e. end stage kidney failure) and the date that the echocardiogram (ECHO) reading was performed on the patient.	Days	0	
3	ECHO_2CLVEDV	Num	2cLVEDV: two-chamber view left ventricular end-diastolic volume	ml		
4	ECHO_2CLVESV	Num	2cLVESV: two-chamber view left ventricular end-systolic volume	ml		
5	ECHO_4CLAD	Num	Four-chamber view long axis diameter	mm		
6	ECHO_4CLVEDV	Num	4cLVEDV: four-chamber view left ventricular end-diastolic volume	ml		
7	ECHO_4CLVESV	Num	4cLVESV: four-chamber view left ventricular end-systolic volume	ml		
8	ECHO_4CSAD	Num	Four-chamber view short axis diameter	mm		
9	ECHO_AOD	Num	Aortic root diameter diastole	mm		
10	ECHO_AVR	Char	Aortic valve regurgitation			
11	ECHO_AVR_DES	Char	Aortic valve regurgitation Description			
12	ECHO_DOPPLER_ADDITIONAL	Num	Additional general evaluation (yes/no)			
13	ECHO_DOPPLER_AORTIC_AOVTI	Num	Aortic Valve: Velocity-time integral	m/sec		
14	ECHO_DOPPLER_AORTIC_EJECTIONTIME	Num	Aortic Valve: Ejection time	msec		
15	ECHO_DOPPLER_AORTIC_LVOTVTI	Num	Aortic Valve: Left ventricular out-flow tract velocity-time integral	m/sec		
16	ECHO_DOPPLER_AORTIC_MG	Num	Aortic mean gradient	mmHg		
17	ECHO_DOPPLER_AORTIC_PG	Num	Aortic peak gradient	mmHg		
18	ECHO_DOPPLER_MITRAL_AWAVE	Num	Mitral Valve: Peak A-wave	m/sec		
19	ECHO_DOPPLER_MITRAL_DT	Num	Mitral Valve: Deceleration time of E-wave	msec		
20	ECHO_DOPPLER_MITRAL_EWAVE	Num	Mitral Valve: Peak E-wave	m/sec		
21	ECHO_DOPPLER_MITRAL_IVRT	Num	Mitral Valve: Isovolumic relaxation time	msec		
22	ECHO_DOPPLER_MITRAL_MG	Num	MG: mean gradient of mitral inflow	mmHg		
23	ECHO_DOPPLER_PHT	Num	PHT: pressure half-time of aortic regurgitation (if present)	msec		
24	ECHO_DOPPLER_TRICUSPID_AWAVE	Num	Tricuspid Valve: Peak A-wave	m/sec		
25	ECHO_DOPPLER_TRICUSPID_EWAVE	Num	Tricuspid Valve: Peak E-wave	m/sec		
26	ECHO_DOPPLER_TRICUSPID_GRADIENT	Num	Tricuspid Valve: Peak gradient of tricuspidal regurgitation	mmHg		
27	ECHO_EA_RATIO_MITRAL	Num	E/A RATIO MITRAL VALVE (yes/no)			
28	ECHO_EA_RATIO_TRICUSPID	Num	E/A RATIO TRICUSPID VALVE (yes/no)			
29	ECHO_EEM_RATIO_MITRAL	Num	E/Em RATIO MITRAL VALVE (yes/no)			
30	ECHO_EET_RATIO_TRICUSPID	Num	E/Et RATIO TRICUSPID VALVE (yes/no)			
31	ECHO_EFSIMPSON	Num	Left ventricular ejection fraction by the Simpson Algorithm	%		
32	ECHO_IVCE	Num	Inferior vena cava diameter espiration	mm		
33	ECHO_IVCI	Num	Inferior vena cava diameter inspiration	mm		
34	ECHO_IVSD	Num	Inter ventricular septum diastole	mm		
35	ECHO_IVSS	Num	IVSs: inter ventricular septum systole	mm		
36	ECHO_LADS	Num	Left atrium diameter systole	mm		
37	ECHO_LAV	Num	Left atrium volume	ml		
38	ECHO_LVDD	Num	Left ventricle diameter diastole	mm		
39	ECHO_LVDS	Num	Left ventricle diameter systole	mm		
40	ECHO_LVESCWS	Num	LEFT VENTRICULAR END-SYSTOLIC CIRCUMFERENTIAL WALL STRESS			
41	ECHO_LVFS	Num	LVFS: LEFT VENTRICULAR FRACTIONAL SHORTENING	%		
42	ECHO_LVM	Num	Left Ventricular Mass	g		
43	ECHO_LVMI_BSA	Num	LVMI: Left Ventricular Mass Index LVMI BSA	g/m^2		
44	ECHO_LVMI_H27	Num	LVMI: Left Ventricular Mass Index LVMI h2.7 (yes/no)			
45	ECHO_LVOT	Num	Left ventricular outflow tract diameter systole	mm		
46	ECHO_LVPWD	Num	Left ventricular posterior wall diastole	mm		
47	ECHO_LVPWS	Num	LVPWs: Left Ventricular Posterior Wall systole	mm		
48	ECHO_MAPSE	Num	MAPSE: Mitral Annular Plane Systolic Excursion	mm		
49	ECHO_MVR	Char	Mitral valve regurgitation			
50	ECHO_MVR_DES	Char	Mitral valve regurgitation Description			
51	ECHO_PERICARDIUM_EFFUSION	Num	Pericardium: pericardial effusion	ml		
52	ECHO_PULSEDDOPPLER_MITRAL_AWAVE	Num	Mitral valve Am: Peak A-wave	m/sec		
53	ECHO_PULSEDDOPPLER_MITRAL_EWAVE	Num	Mitral valve Em: Peak E-wave	m/sec		
54	ECHO_PULSEDDOPPLER_MITRAL_SWAVE	Num	Mitral valve Sm: Peak S-wave	m/sec		

55	ECHO_PULSEDDOPPL_TRICUSPID_AWAVE	Num	Tricuspid valve Am: Peak A-wave	m/sec
56	ECHO_PULSEDDOPPL_TRICUSPID_EWAVE	Num	Tricuspid valve Em: Peak E-wave	m/sec
57	ECHO_PULSEDDOPPL_TRICUSPID_SWAVE	Num	Tricuspid valve Sm: Peak S-wave	m/sec
58	ECHO_RAV	Num	RAV: Right Atrium Volume	ml
59	ECHO_RVDD	Num	Right ventricle diameter diastole	mm
60	ECHO_RWT	Num	RWT: Relative Wall Thickness (yes/no)	
61	ECHO_SBP	Num	Last monthly average SBP	mmHg
62	ECHO_TAPSE	Num	Tricuspid annular plane systolic excursion	mm
63	ECHO_TVR	Char	Tricuspid valve regurgitation	
64	ECHO_TVR_DES	Char	Tricuspid valve regurgitation Description	
65	ECHO_VCF	Num	VCF: LV mean Velocity of Circumferential Fibre Shortening	

Event_DB

Number	Variable	Variable Type	Description	Unit	Cleaning rules Min valid value	Cleaning rules Max valid value
1	GFME_ID	Num	**Primary key**: Randomly assigned patient identification number.			
2	DAYS_FROM_FDD	Num	Days between the first date of dialysis (FDD) for kidney failure (i.e. end stage kidney failure) and the date of the "event". In this table, the "events" include death, hospitalization, vascular access removal, modality change, transplantation, withdrawal from dialysis, recovery, change of clinic/provider, and peritonitis events.	Days	0	
3	ACCESS_REMOVAL_REASON	Char	Dialysis access removal reason. This is the first / primary reason for the dialysis access being removed for a unique patient on a specific day from the first date of dialysis (FDD). This applies to all access types for all modalities (e.g. AVF, AVG, CVC, PD catheter).	Text category		
4	COD_CUSTOMTEXT	Char	Text category for the first / primary cause of death (COD).	Text category		
5	COD_ICD10TEXT	Char	ICD 10 code for the first / primary cause of death (COD).	ICD 10 code		
6	COH_ICD10TEXT1	Char	ICD 10 code for the first / primary cause of hospitalization (COH). This is the primary diagnosis reason for the hospitalization determined at discharge. This data is reported on the row denoting the days from first date of dialysis (FDD) that the patient was admitted into in-patient care starting a hospitalization event.	ICD 10 code		
7	EVENT_ACCESS_REMOVAL	Num	Denotation if the dialysis access was removed (EVENT_ACCESS_REMOVAL=1). This will mark if the dialysis access was removed for a unique patient on a specific day from the first date of dialysis (FDD). This applies to all access types for all modalities (e.g. AVF, AVG, CVC, PD catheter).			
8	EVENT_DIED	Num	Denotation if the patient died (EVENT_DIED=1). This will mark the days from the first date of dialysis (FDD) that the patient had a death event.			
9	EVENT_DISCHARGE	Num	Denotation if the patient was discharged from the dialysis clinic (EVENT_DISCHARGE=1). This will mark the days from the first date of dialysis (FDD) that the patient was discharged from the clinic for one of the predefined categorical reasons denoted in the associated field EVENT_DISCHARGE_REASON.			
10	EVENT_DISCHARGE_REASON	Char	Categorized reason patient was discharged from the dialysis clinic.			
11	EVENT_HOSP	Num	Denotation if the patient was admitted to the hospital (EVENT_HOSP=1). This will mark the days from the first date of dialysis (FDD) that the patient was admitted as an in-patient at the hospital.			
12	HOSP_DAYS	Num	Number of hospital days from the date of admission to the date of discharge from the hospital. This will represent the length of stay for the hospitalization. This data is reported on the row denoting the days from first date of dialysis (FDD) that the patient was admitted into in-patient care starting a hospitalization event.	Days	1	
13	COH_ICD10TEXT2	Char	ICD 10 code for the second / secondary cause of hospitalization (COH). This is the secondary diagnosis reason for the hospitalization determined at discharge. This data is reported on the row denoting the days from first date of dialysis (FDD) that the patient was admitted into in-patient care starting a hospitalization event.	ICD 10 code		
14	COH_ICD10TEXT3	Char	ICD 10 code for the third / tertiary cause of hospitalization (COH). This is the tertiary diagnosis reason for the hospitalization determined at discharge. This data is reported on the row denoting the days from first date of dialysis (FDD) that the patient was admitted into in-patient care starting a hospitalization event.	ICD 10 code		
15	EVENT_PERITONITIS	Num	Denotation if patient had a peritonitis infection (EVENT_PERITONITIS=1). This will mark the days from the first date of dialysis (FDD) that a peritoneal dialysis (PD) patient was determined to have a peritonitis infection.			

Lab_DB

Number	Variable	Variable Type	Description	Unit	Cleaning rules Min valid value	Cleaning rules Max valid value
1	GFME_ID	Num	**Primary key**: Randomly assigned patient identification number.			
2	DAYS_FROM_FDD	Num	Days between the first date of dialysis (FDD) for kidney failure (i.e. end stage kidney failure) and the date that the laboratory was collected from the patient. Each row will include all lab values for a unique patient that occurred on that number of days from the FDD. If two valid results were reported for the same lab on the same date (expressed as days from FDD), this is being report on a separate row.	Days	0	
3	LAB_ACID_PHOSPHATASE	Num	Acid phosphatase lab value for a patient for each unique specimen collection.	U/L		
4	LAB_ALBUMIN	Num	Albumin lab value for a patient for each unique specimen collection.	g/dL	1.5	6
5	LAB_AFP	Num	Alpha-fetoprotein (AFP) lab value for a patient for each unique specimen collection.	U/mL		
6	LAB_ALK_PHOSPH	Num	Alkaline phosphatase (ALP) lab value for a patient for each unique specimen collection.	U/L	5	3500
7	LAB_ALT	Num	Alanine transaminase (ALT) lab value for a patient for each unique specimen collection.	U/L	3	5500
8	LAB_ALUMINIUM	Num	Aluminium lab value for a patient for each unique specimen collection.	µg/l	0	1000
9	LAB_AMYLASE	Num	Amylase lab value for a patient for each unique specimen collection.	U/L	10	2000
10	LAB_AMMONIA	Num	Ammonia lab value for a patient for each unique specimen collection.	µg/dL		
11	LAB_ANION_GAP	Num	Anion Gap lab value for a patient for each unique specimen collection.	mmol/L		
12	LAB_AST	Num	Aspartate aminotransferase (AST) lab value for a patient for each unique specimen collection.	U/L	3	5500
13	LAB_BASOPHILS	Num	Basophil count as a percent of the white blood cells for a patient for each unique specimen collection. This is generally part of the complete blood count (CBC) lab that includes a white blood differential count.	%	0	15
14	LAB_BETA2	Num	Beta-2-microglobulin lab value for a patient for each unique specimen collection.	mg/L	0.5	130
15	LAB_BICARB	Num	Bicarbonate lab value for a patient for each unique specimen collection. This is the pre-dialysis value for patients treated incenter (HD, HDF, …). Bicarbonate values for peritoneal dialysis patients are entered into this field.	mg/L	2	45
16	LAB_BICARB_POST	Num	Post-dialysis bicarbonate lab value for a patient for each unique specimen collection. This field is specifically for patients with post-dialysis laboratories drawn (e.g. incenter HD, HDF, …). This field is not applicable for peritoneal dialysis patients.	mg/L	2	45
17	LAB_BILIRUBIN_TOTAL	Num	Bilirubin lab value for a patient for each unique specimen collection.	mg/dL	0	72
18	LAB_BLOOD_TYPE	Num	Blood type lab for a patient for each unique specimen collection. Blood type includes ABO grouping and Rho(D) typing if the lab is available.			
19	LAB_BNP	Num	Brain natriuretic peptide (BNP) lab value for a patient for each unique specimen collection.	pg/mL		
20	LAB_C3	Num	Complement C3 lab value for a patient for each unique specimen collection.	mg/dL		
21	LAB_C4	Num	Complement C4 lab value for a patient for each unique specimen collection.	mg/dL		
22	LAB_CA_125	Num	Cancer antigen 125 (CA 125) lab value for a patient for each unique specimen collection.	U/mL		
23	LAB_CA_15_3	Num	Cancer antigen 15-3 (CA 15-3) lab value for a patient for each unique specimen collection.	U/mL		
24	LAB_CA_19_9	Num	Cancer antigen 19-9 (CA 19-9) lab value for a patient for each unique specimen collection.	U/mL		
25	LAB_CA_50	Num	Cancer Antigen 50 (CA-50) lab value for a patient for each unique specimen collection.	U/mL		
26	LAB_CALCIUM	Num	Total uncorrected calcium lab value for a patient for each unique specimen collection. This is the pre-dialysis value for patients treated incenter (HD, HDF, …). Calcium values for peritoneal dialysis patients are entered into this field.	mg/dL	4	18
27	LAB_CALCIUM_IONIZED	Num	Ionized calcium lab value for a patient for each unique specimen collection.	mg/dL	1.16	13.28
28	LAB_CALCIUM_POST	Num	Post-dialysis total uncorrected calcium lab value for a patient for each unique specimen collection. This field is specifically for patients with post-dialysis laboratories drawn (e.g. incenter HD, HDF, …). This field is not applicable for peritoneal dialysis patients.	mg/dL	4	18
29	LAB_CEA	Num	Carcinoembryonic Antigen (CEA) lab value for a patient for each unique specimen collection.	ng/mL		
30	LAB_CHLORIDE	Num	Chloride lab value for a patient for each unique specimen collection.	mEq/L	50	200
31	LAB_CHOLESTEROL	Num	Cholesterol lab value for a patient for each unique specimen collection.	mg/dL	25	800
32	LAB_CHOLINESTERASE_CHE	Num	Cholinesterase (CHE) lab value for a patient for each unique specimen collection.	U/L		
33	LAB_CREATINE_KINASE	Num	Creatine kinase (CK) lab value for a patient for each unique specimen collection.	U/L	10	1500000
34	LAB_CREATINE_KINASE_MB	Num	Creatine kinase myocardial band (CK-MB) lab value for a patient for each unique specimen collection.	U/L		
35	LAB_CREATININE	Num	Creatinine lab value for a patient for each unique specimen collection.	mg/dL	0.2	55
36	LAB_CRP	Num	C-reactive protein (CRP) lab value for a patient for each unique specimen collection.	mg/L	0.1	625
37	LAB_WKTV	Num	Total weekly Kt/V lab calculation value for a home dialysis (PD) patient for each unique specimen collection.		0.4	8
38	LAB_EKTV	Num	Equilibrated Kt/V lab value for a patient for each unique specimen collection. In Fresenius Medical Care in the United States, this is calculated using the Tattersall equation.		0.4	8
39	LAB_SPKTV	Num	Single-pool Kt/V lab value for a patient for each unique specimen collection. This is calculated using the Daugirdas equation.		0.4	8
40	LAB_EOSINOPHILS	Num	Eosinophil count as a percent of the white blood cells for a patient for each unique specimen collection. This is generally part of the complete blood count (CBC) lab that includes a white blood differential count.	%	0	20
41	LAB_ESR	Num	Erythrocyte sedimentation rate (ESR) lab value for a patient for each unique specimen collection.	mm/h	0	250

42	LAB_FERRITIN	Num	Ferritin lab value for a patient for each unique specimen collection.	ng/mL	2	200000
43	LAB_FIBRINOGEN	Num	Fibrinogen lab value for a patient for each unique specimen collection.	mg/dL		
44	LAB_FOLIC_ACID	Num	Folic acid lab value for a patient for each unique specimen collection.	ng/mL	0	100
45	LAB_FRUCTOSAMINE	Num	Fructosamine lab value for a patient for each unique specimen collection.	µmol/L	25	800
46	LAB_FT3	Num	Free triiodothyronine (FT3) lab value for a patient for each unique specimen collection.	pg/dL	0	150
47	LAB_FT4	Num	Free thyroxine (FT4) lab value for a patient for each unique specimen collection.	ng/dL	0.1	12
48	LAB_GGT	Num	Gamma Glutamyl Transferase (GGT) lab value for a patient for each unique specimen collection.	U/L	0	2000
49	LAB_GLUCOSE	Num	Glucose lab value for a patient for each unique specimen collection.	mg/dL	18	3000
50	LAB_GLUTAMATE_DEHYDROGENASE_GLD	Num	Glutamate Dehydrogenase (GLD) lab value for a patient for each unique specimen collection.	U/L		
51	LAB_GLYCEMIC_08	Num	Glycemic measurement (08:00)	mg/dL		
52	LAB_GLYCEMIC_11	Num	Glycemic measurement (11:00)	mg/dL		
53	LAB_GLYCEMIC_17	Num	Glycemic measurement (17:00)	mg/dL		
54	LAB_PT	Num	Prothrombin Time (PT) lab value for a patient for each unique specimen collection.	seconds		
55	LAB_HAPTAGLOBIN	Num	Haptoglobin lab value for a patient for each unique specimen collection.	mg/dL		
56	LAB_HBC_AC	Char	Ac anti-HBc result for a patient for each unique specimen collection.			
57	LAB_HBC_ANTIHBS	Num	Anti-HBs measurement for a patient for each unique specimen collection.	U/L		
58	LAB_HBC_HBSAG	Char	Hbs Ag result for a patient for each unique specimen collection.			
59	LAB_HBE_AC	Char	Ac anti-HBe result for a patient for each unique specimen collection.			
60	LAB_HBE_AG	Char	Ag HBe result for a patient for each unique specimen collection.			
61	LAB_HBSAB_STATUS	Num	Hepatitis B virus surface antibody (HBsAb) lab categorical result for a patient for each unique specimen collection.			
62	LAB_HCT	Num	Hematocrit lab value for a patient for each unique specimen collection. This is part of the complete blood count (CBC).	%	1	85
63	LAB_HCV_ELISA_Status	Char	HCV Status Code Hepatitis C virus (HCV) enzyme-linked immunosorbent assay (ELISA) result for a patient for each unique specimen collection.			
64	LAB_HCV_RT_PCR	Char	Hepatitis C virus RNA polymerase chain reaction (PCR) result for a patient for each unique specimen collection.			
65	LAB_HCV_RIBA_Status	Char	Hepatitis C virus (HCV) recombinant immunoblot assay (RIBA) quantitative antibody result for a patient for each unique specimen collection.			
66	LAB_HDL	Num	High-density lipoprotein (HDL) lab value for a patient for each unique specimen collection.	mg/dL	0	250
67	LAB_HEP_B_AB_IGG	Char	Hep b ab igg result			
68	LAB_HGB	Num	Hemoglobin (Hgb) lab value for a patient for each unique specimen collection. This is part of the complete blood count (CBC).	g/dL	0.6	30
69	LAB_HGBA1C	Num	Hemoglobin A1C (HgbA1C) lab value for a patient for each unique specimen collection. This is generally for patients with diabetes. Percentage of total hemoglobin.	%	3	15
70	LAB_IL_8	Num	Interleukin-8 (IL-8) lab value for a patient for each unique specimen collection.	ng/L		
71	LAB_IL6	Num	Interleukin-6 (IL-6) lab value for a patient for each unique specimen collection.	ng/L		
72	LAB_INR	Num	International normalized ratio (INR) lab value for a patient for each unique specimen collection.		0	20
73	LAB_IRON	Num	Total iron lab value for a patient for each unique specimen collection.	mcg/dL	5	5000
74	LAB_LACTATE_DEHYDROGENASE_LD	Num	Lactate Dehydrogenase (LD) lab value for a patient for each unique specimen collection.	U/L		
75	LAB_LDL	Num	Low-density lipoprotein (LDL) lab value for a patient for each unique specimen collection.	mg/dL	7	400
76	LAB_LEAD	Num	Lead lab value for a patient for each unique specimen collection.	µg/L		
77	LAB_LIPASE	Num	Lipase lab value for a patient for each unique specimen collection.	U/L	0	1900
78	LAB_LYMPHOCYTES	Num	Lymphocyte count as a percent of the white blood cells for a patient for each unique specimen collection. This is generally part of the complete blood count (CBC) lab that includes a white blood differential count.	%	0	100
79	LAB_MERCURY	Num	Mercury lab value for a patient for each unique specimen collection.	µg/L		
80	LAB_MG	Num	Magnesium (Mg) lab value for a patient for each unique specimen collection.	mg/dL	0	10
81	LAB_MONOCYTES	Num	Monocyte count as a percent of the white blood cells for a patient for each unique specimen collection. This is generally part of the complete blood count (CBC) lab that includes a white blood differential count.	%	0	100
82	LAB_MYELOCYTES	Num	Myelocytes as a percent of the white blood cells for a patient for each unique specimen collection. This may not be common, but can be part of a complete blood count (CBC) with differential.	%	0.1	100
83	LAB_MYOGLOBIN	Num	Myoglobin lab value for a patient for each unique specimen collection.	µg/L		
84	LAB_NEUTROPHILS	Num	Neutrophils as a percent of the white blood cells for a patient for each unique specimen collection. This is generally part of the complete blood count (CBC) lab that includes a white blood differential count.	%	0	100
85	LAB_NPCR	Num	Normalized protein catabolic rate (nPCR) lab value for a patient for each unique specimen collection.	g/kg/day	0.01	100
86	LAB_OSTEOCALCIN	Num	Osteocalcin lab value for a patient for each unique specimen collection.	ng/mL		
87	LAB_PCO2	Num	Partial pressure of carbon dioxide (PCO2) blood gas lab value for a patient for each unique specimen collection.	mmHg		
88	LAB_PH	Num	Potential of hydrogen (pH) measurement for a patient for each unique specimen collection.		0	14
89	LAB_PHOSPH	Num	Phosphate (Phosph, Phos, or PO4) lab value for a patient for each unique specimen collection. This is the pre-dialysis value for patients treated incenter (HD, HDF, …). Phosphate values for peritoneal dialysis patients are entered into this field.	mg/dL	0.5	20

90	LAB_PHOSPH_POST	Num	Post-dialysis Phosphate (Phosph, Phos, or PO4) lab value for a patient for each unique specimen collection. This field is specifically for patients with post-dialysis laboratories drawn (e.g. incenter HD, HDF, ...). This field is not applicable for peritoneal dialysis patients.	mg/dL	0.5	20
91	LAB_PLATELETS	Num	Platelet count lab value for a patient for each unique specimen collection. This is part of the complete blood count (CBC).	10^3/µL	4	3000
92	LAB_PLATELETS_VOLUME	Num	Mean platelet volume as the average size of the platelets (femtoliters (fL) per cell) in the blood for a patient for each unique specimen collection. This can be part of the complete blood count (CBC).	fL	2	26
93	LAB_PO2	Num	Partial pressure of oxygen (PO2) blood gas lab value for a patient for each unique specimen collection.	mmHg		
94	LAB_POTASSIUM_POST	Num	Post-dialysis Potassium (K) lab value for a patient for each unique specimen collection. This field is specifically for patients with post-dialysis laboratories drawn (e.g. incenter HD, HDF, ...). This field is not applicable for peritoneal dialysis patients.	mEq/L	1	20
95	LAB_PREALBUMIN	Num	Prealbumin lab value for a patient for each unique specimen collection.	mg/dL	1	100
96	LAB_PROCALCITONIN	Num	Procalcitonin lab value for a patient for each unique specimen collection.	ng/mL		
97	LAB_PROTEIN_PROFILE_ALFA1	Num	Alpha-1 globulin lab value for a patient for each unique specimen collection.	g/dL		
98	LAB_PROTEIN_PROFILE_ALFA1_CALC	Num	Alpha-1 globulin % of normal lab value for a patient for each unique specimen collection.	%		
99	LAB_PROTEIN_PROFILE_ALFA2	Num	Alpha-2 globulin lab value for a patient for each unique specimen collection.	g/dL		
100	LAB_PROTEIN_PROFILE_BETA	Num	Beta globulin lab value for a patient for each unique specimen collection.	g/dL		
101	LAB_PROTEIN_PROFILE_BETA1	Num	Beta-1 globulin lab value for a patient for each unique specimen collection.	g/dL		
102	LAB_PSA	Num	Prostate-specific antigen (PSA) lab value for a patient for each unique specimen collection.	µg/L	0	8000
103	LAB_APTT	Num	Activated partial thromboplastin time (aPTT) lab value for a patient for each unique specimen collection.	s (seconds)	0	120
104	LAB_RBC_COUNT	Num	Red blood cell (RBC) count lab value for a patient for each unique specimen collection. This is part of the complete blood count (CBC).	10^6/µL	0	8
105	LAB_RBC_DW	Num	Red Blood Cell Distribution Width (RBC-DW) as a percent variation in the volume and size of RBCs for a patient for each unique specimen collection. This can be part of the complete blood count (CBC) lab that includes a white blood differential count.	%	0.1	100
106	LAB_RETICULOCYTES	Num	Reticulocyte count as a percent of the red blood cells for a patient for each unique specimen collection. This can be part of the complete blood count (CBC) lab that includes a blood differential count.	%	0.1	24.5
107	LAB_SERUM_AMYLOID_A_SAA	Num	Serum amyloid A (SAA) lab value for a patient for each unique specimen collection.	mg/L		
108	LAB_SERUM_NA_POST	Num	Post-dialysis sodium (Na) lab value for a patient for each unique specimen collection. This field is specifically for patients with post-dialysis laboratories drawn (e.g. incenter HD, HDF, ...). This field is not applicable for peritoneal dialysis patients.	mEq/L	50	200
109	LAB_TIBC	Num	Total iron binding capacity (TIBC) lab value for a patient for each unique specimen collection.	µg/dL	10	1000
110	LAB_TOTAL_PROTEIN	Num	Total protein lab value for a patient for each unique specimen collection.	g/dL	3	20
111	LAB_TRANSFERRIN	Num	Transferrin lab value for a patient for each unique specimen collection.	mg/dL	75	750
112	LAB_TRIGLYCERIDES	Num	Triglycerides lab value for a patient for each unique specimen collection.	mg/dL	10	5000
113	LAB_TROPONIN	Num	Troponin T lab value for a patient for each unique specimen collection.	ng/mL	0	150
114	LAB_TSH	Num	Thyroid-stimulating hormone (TSH) lab value for a patient for each unique specimen collection.	µIU/mL	0.01	1300
115	LAB_TT3	Num	Total triiodothyronine (TT3) lab value for a patient for each unique specimen collection.	ng/mL	0.1	8
116	LAB_URIC_ACID	Num	Uric acid lab value for a patient for each unique specimen collection.	mg/dL	1.5	30
117	LAB_URR	Num	Urea Reduction Ratio (URR) lab value calculation for a patient for each unique specimen collection.	%	0	100
118	LAB_VITAMIN_B12	Num	Vitamin B12 lab value for a patient for each unique specimen collection.	pg/mL	45	2000
119	LAB_VITAMIN_D125	Num	Vitamin D 1,25 (also known as Vitamin D3, 1,25-dihydroxyvitamin D , 1,25-dihydroxycholecalciferol, 1,25-dihydroxyvitamin D3) lab value for a patient for each unique specimen collection.	pg/mL	2	250
120	LAB_WBC	Num	White blood cell (WBC) count lab value for a patient for each unique specimen collection. This is part of the complete blood count (CBC).	10^3/µL	0	400
121	URINE_GLUCOSE	Num	Urine glucose lab value for a patient for each unique specimen collection.	mmol/L		
122	URINE_OSMOLARITY	Num	Urine osmolarity lab value for a patient for each unique specimen collection.	mmol/kg		
123	URINE_POTASSIUM	Num	Urine potassium lab value for a patient for each unique specimen collection.	mmol/24h		
124	URINE_PROTEIN	Num	Urine protein lab value for a patient for each unique specimen collection.	mg/dL	0	19000
125	URINE_SODIUM	Num	Urine sodium lab value for a patient for each unique specimen collection.	mmol/24h		
126	URINE_BICARBONATE	Num	Bicarbonate lab value for a patient for each unique specimen collection.	mmol/L		
127	URINE_MICROALBUMINARIA	Num	Urine albumin lab value for a patient for each unique specimen collection.	mg/24h		
128	LAB_POTASSIUM	Num	Potassium (K) lab value for a patient for each unique specimen collection. This is the pre-dialysis value for patients treated incenter (HD, HDF, ...). Potassium values for peritoneal dialysis patients are entered into this field.	mEq/L	1	20
129	LAB_SERUM_NA	Num	Sodium (Na) lab value for a patient for each unique specimen collection. This is the pre-dialysis value for patients treated incenter (HD, HDF, ...). Sodium values for peritoneal dialysis patients are entered into this field.	mEq/L	50	200
130	LAB_TT4	Num	Total thyroxine (TT4) lab value for a patient for each unique specimen collection.	µg/dL	0.3	30

131	LAB_BUN_POST	Num	Post-dialysis blood urea nitrogen (BUN) lab value for a patient for each unique specimen collection. This field is specifically for patients with post-dialysis laboratories drawn (e.g. incenter HD, HDF, …). This field is not applicable for peritoneal dialysis patients.	mg/dL	1	250
132	LAB_BUN_PRE	Num	Blood urea nitrogen (BUN) lab value for a patient for each unique specimen collection. This is the pre-dialysis value for patients treated incenter (HD, HDF, …). BUN values for peritoneal dialysis patients are entered into this field.	mg/dL	1	250
133	LAB_CALCIUM_CORRECTED	Num	Corrected calcium lab value for a patient for each unique specimen collection.	mg/dL		
134	LAB_PTH	Num	Intact Parathyroid hormone (PTH) lab value for a patient for each unique specimen collection.	ng/L	2.5	2500
135	LAB_MCH	Num	Mean Corpuscular Hemoglobin (MCH) as the mass of hemoglobin (pg) per red blood cell for a patient for each unique specimen collection. This can be part of the complete blood count (CBC) with blood differential.	pg/cell	5	50
136	LAB_MCHC	Num	Mean corpuscular hemoglobin concentration (MCHC) as the mass of hemoglobin (g) per volume of packed red blood cells (dL) for a patient for each unique specimen collection. This can be part of the complete blood count (CBC) with blood differential.	g/dL	10	100
137	LAB_MCV	Num	Mean corpuscular volume (MCV) as the average size of the red blood cells (femtoliters (fL) per cell) for a patient for each unique specimen collection. This can be part of the complete blood count (CBC) with blood differential.	fL	30	200
138	LAB_TSAT	Num	Transferrin saturation (TSAT) lab value for a patient for each unique specimen collection.	%	0.1	100
139	URINE_UREA_CLEARANCE	Num	Urine urea clearance lab calculation for a patient for each unique specimen collection.	mL/min	0	500
140	URINE_CREATININE	Num	Urine creatinine lab value for a patient for each unique specimen collection.	mg/dL	0	1100
141	URINE_CREATININE_CLEARANCE	Num	Urine creatinine clearance lab calculation for a patient for each unique specimen collection.	mL/min	0	800
142	URINE_VOLUME	Num	Urine volume per 24 hours for a patient for each unique specimen collection. This is the volume of urine output produced in a 24 hour urine specimen collection. This volume may be needed for determining the concentration of some urine results that are reported as a quantity per 24 hours.	mL	0	10000
143	URINE_UREA	Num	Urine urea lab value for a patient for each unique specimen collection.	mg/dL	10	2000
144	LAB_SARS_COV_2_SUSPECTED	Num	This field documents each time a patient was suspected of having SARS-COV-2 infection (as of a specific number of days from the first date of dialysis).			
145	LAB_SARS_COV_2_CONFIRMED	Num	This field documents each time a patient was confirmed positive for SARS-COV-2 (as of a specific number of days from the first date of dialysis). Confirmation of SARS-COV-2 infection includes any type of laboratory/point of care testing.			
146	LAB_DPKTV	Num	Total weekly Kt/V lab calculation value for a peritoneal dialysis (PD) patient for each unique specimen collection. This lab uses PD effluent fluid and urine. This field should only have data for PD patients.		0.4	8
147	LAB_HBSAB	Num	Hepatitis B virus surface antibody (HBsAb) lab value for a patient for each unique specimen collection.	m[IU]/mL		
148	LAB_HCV_PCR	Num	Hepatitis C virus (HCV) polymerase chain reaction (PCR) quantitative RNA lab value for a patient for each unique specimen collection.	[IU]/mL		
149	LAB_SARS_COV_2_PCR	Num	This field documents if a patient was confirmed to be positive for SARS-COV-2 by a PCR testing method. This field is intended to only be for patients confirmed to have SARS-COV-2 infection as documented in the field "LAB_SARS_COV_2_CONFIRMED". 1= PCR test was used, 0=non-PCR test was used (e.g. antigen test)			
150	LAB_VITAMIN_D25	Num	Vitamin D25 (also known as 25-hydroxy vitamin D, 25-OH Vitamin D) lab value for a patient for each unique specimen collection.	ng/mL	4	150

TXT_DB

Number	Variable	Variable Type	Description	Unit	Cleaning rules Min valid value	Cleaning rules Max valid value
1	GFME_ID	Num	**Primary key**: Randomly assigned patient identification number.			
2	DAYS_FROM_FDD	Num	Days between the first date of dialysis (FDD) for kidney failure (i.e. end stage kidney failure) and the date of the "event". In this table, the "event" is a dialysis treatment and any associated data for the fields in this table.	Days		
3	ACCESS_LOCATION	Char	Dialysis access location. Field can include anatomical position on body and/or artery and vein used for access. Peritoneal dialysis catheter location data are omitted from this field, and entered in "ACCESS_QUADRANT".			
4	ACCESS_QUADRANT	Char	Quadrant of peritoneal dialysis (PD) catheter.			
5	ACCESS_TYPE	Char	Dialysis access type.			
6	COUNTRY	Char	Country where patient received dialysis.			
7	FACILITY_ID	Num	**Primary key**: Randomly assigned clinic identification number.			
8	TXT_ARTERIAL_PRESSURE	Num	Arterial pressure measured from the hemodialysis machine (typically pre-pump arterial pressure on hemodialysis machines).	mmHg	-400	400
9	TXT_ART_NEEDLE_SIZE	Char	Size of the arterial hemodialysis needle (gauge or mm) and/or length of arterial needle (inch or cm)			
10	TXT_AUTOFLOW_ENABLED	Char	Whether AutoFlow for the dialysate flow is enabled on hemodialysis machine (TXT_AUTOFLOW_ENABLED=1). AutoFlow feature allows the dialysate flow rate to be automatically set to a standardized ratio above the blood flow rate.			
11	TXT_BVM_RBV_CRITICAL	Num	Relative blood volume (RBV) critical levels are calculated by blood volume monitor (BVM) hemodialysis and hemodiafiltration machines. RBV critical level is defined as the percent change in RBV (from the start of HD/HDF) at which a patient develops symptomatic hypotension. BVM device is typically Blood Volume Monitor built into machine or a separate Crit-Line device installed in the dialysis circuit.	%	0	100
12	TXT_BVM_UF_MAX	Num	Blood volume monitor (BVM) calculated maximum ultrafiltration (UF) rate. Calculation is based on volume entered at beginning of hemodialysis treatment, and maximum UF is 2 times the total UF divided by dialysis time remaining in dialysis.	mL/Hour		
13	TXT_DIALYSIS_MACHINE	Char	Hemodialysis machine, hemodiafiltration machine, home hemodialysis machine, or automated peritoneal dialysis (APD) machine used.			
14	TXT_DIALYZER	Char	Dialyzer type used for hemodialysis or hemodiafiltration.			
15	TXT_DIAL_BICARB	Num	Prescribed dialysate bicarbonate concentration.	mEq/L	0	45
16	TXT_DIAL_CALCIUM	Num	Prescribed dialysate calcium concentration.	mEq/L	0	5
17	TXT_DIAL_DEXTROSE	Char	Prescribed dialysate dextrose concentration.	mg/dL or %		
18	TXT_DIAL_K	Num	Prescribed dialysate potassium concentration.	mEq/L	0	5
19	TXT_DIAL_MG	Num	Prescribed dialysate magnesium concentration.	mEq/L	0	5
20	TXT_DIAL_NA	Num	Prescribed dialysate sodium concentration.	mEq/L	110	175
21	TXT_DIAL_TEMP	Num	Prescribed dialysate temperature.	Celsius	25	45
22	TXT_DRY_WEIGHT	Num	Estimated dry weight. This is estimated weight of the patient without excess fluid. Generally, this is determined by the physician considering the lowest weight tolerated without the development of intradialytic symptoms.	Kg	20	600
23	TXT_EDEMA	Char	Findings from the assessment of edema including the pitting and location, as applicable. Can comma separate pitting and location as needed if combining fields (pitting, location).			
24	TXT_END_TIME	Char	This is the local time the hemodialysis or hemodiafiltration treatment ended, per the report from the dialysis machine or the charted time in the medical record.	hh:mm:ss	00:00:00	23:59:59
25	TXT_INTRA_DBP_MAX	Num	Maximum intradialytic diastolic blood pressure (DBP). This is the highest DBP reading that was recorded during each hemodialysis or hemodiafiltration treatment.	mmHg	5	400
26	TXT_INTRA_DBP_MIN	Num	Minimum intradialytic diastolic blood pressure (DBP). This is the lowest DBP reading that was recorded during each hemodialysis or hemodiafiltration treatment.	mmHg	5	400
27	TXT_INTRA_PULSE_MAX	Num	Maximum intradialytic pulse (i.e. heart rate). This is the highest pulse reading that was recorded during each hemodialysis or hemodiafiltration treatment.	beats/min	5	600
28	TXT_INTRA_PULSE_MIN	Num	Minimum intradialytic pulse (i.e. heart rate). This is the lowest pulse reading that was recorded during each hemodialysis or hemodiafiltration treatment.	beats/min	5	600
29	TXT_INTRA_SBP_MAX	Num	Maximum intradialytic systolic blood pressure (SBP). This is the highest SBP reading that was recorded during each hemodialysis or hemodiafiltration treatment.	mmHg	5	400
30	TXT_INTRA_SBP_MIN	Num	Minimum intradialytic systolic blood pressure (SBP). This is the lowest SBP reading that was recorded during each hemodialysis or hemodiafiltration treatment.	mmHg	5	400
31	TXT_INTRA_UFR_MAX	Num	Maximum ultrafiltration rate (UFR). This is the highest UFR recorded during each hemodialysis or hemodiafiltration treatment.	mL/hour	0	3500
32	TXT_KTV_OCM	Num	Kt/V adequacy for each treatment based on the dialysis machine's online clearance monitor (OCM). This is the final value of kt/V for each treatment. This kt/V is calculated by the OCM using ionic dialysance to measure clearance (K), treatment time (t), and the kinetic modeling of urea distribution volume (V).		0.4	8
33	TXT_LOCATION	Char	Location where the dialysis treatment occurred, either at home or at the in-center/outpatient clinic			

34	TXT_MODALITY	Char	Dialysis modality used for the kidney replacement therapy for each treatment ("HD" = hemodialysis, "HDF" = hemodiafiltration, Mixed HD/HDF, "HF" = hemofiltration, "CAPD" = continuous ambulatory peritoneal dialysis, "CCPD" = continuous cycling peritoneal dialysis/automated peritoneal dialysis (inclusive of long or short daytime dwells), "Tidal" = tidal CCPD (incomplete drain before refill), "IPD" = intermittent CCPD (no day/night dwell - dry by day/night), Other).			
35	TXT_NA_PROFILE	Num	The sodium (Na+) profiling used for a hemodialysis or hemodiafiltration treatment. This is the sodium variation system profile set on the machine for each treatment.			
36	TXT_PD_DWELL_TIME_DAY	Char	Peritoneal dialysis: prescribed dwell time during the daytime. This is the time the peritoneal dialysate solution is prescribed to remain in the abdomen between exchanges during the day.	Minutes	1	1500
37	TXT_PD_EXCHG_NUM_DAY	Num	Peritoneal dialysis: number of exchanges of the dialysate during the daytime. For CCPD, this can be number of cycles if using cycler during the daytime.		0	20
38	TXT_PD_INFLOW_VOL_DAY	Num	Peritoneal dialysis: prescribed inflow volume in the daytime. This is the volume of dialysate solution prescribed to go into abdomen for the daytime dwell(s).	mL	1	10000
39	TXT_PD_TOTAL_VOLUME	Num	Peritoneal dialysis: total prescribed inflow volume per 24 hours. This is the total amount of volume of dialysate solution prescribed to go into abdomen across all dwells in a 24 hour day/period.<br>Note: TXT_PD_TOTAL_VOLUME = (TXT_PD_INFLOW_VOL_DAY * TXT_PD_EXCHG_NUM_DAY) + (TXT_PD_INFLOW_VOL_NIGHT * TXT_PD_EXCHG_NUM_NIGHT)	mL	1	20000
40	TXT_PER_WEEK	Num	Number of dialysis treatments prescribed per week. For PD, this is typically 7 days per week (i.e. daily). This field includes a number from 1 through 7. If patients are treated every other day, this can be shown as "3.5", which will imply the treatment occurs every other day (e.g. every 3 to 4 days depending on the patient's last treatment).			
41	TXT_POST_DBP	Num	Post-dialysis sitting diastolic blood pressure (DBP) vital sign measurement. This includes data from patients treated in dialysis chairs.<br>Note: Peritoneal dialysis patients will not have any data entered in this field and vital sign measurements are only captured in pre-dialysis fields.	mmHg	5	400
42	TXT_POST_DBP_LYING	Num	Post-dialysis lying diastolic blood pressure (DBP) vital sign measurement. This is only for clinics who have patients lying on a bed for dialysis treatments. Patients who are treated in dialysis chairs are not anticipated to have data on lying blood pressure measurements.<br>Note: Peritoneal dialysis patients will not have any data entered in this field and vital sign measurements are only captured in pre-dialysis fields.	mmHg	5	400
43	TXT_POST_PULSE	Num	Post-dialysis pulse (i.e. heart rate) measurement. This is anticipated to be the measurement recorded while the patient was sitting or lying.<br>Note: Peritoneal dialysis patients will not have any data entered in this field and vital sign measurements are only captured in pre-dialysis fields.	beats/min	5	600
44	TXT_POST_SBP	Num	Post-dialysis sitting systolic blood pressure (SBP) vital sign measurement. This includes data from patients treated in dialysis chairs.<br>Note: Peritoneal dialysis patients will not have any data entered in this field and vital sign measurements are only captured in pre-dialysis fields.	mmHg	5	400
45	TXT_POST_SBP_LYING	Num	Post-dialysis lying systolic blood pressure (SBP) vital sign measurement. This is only for clinics who have patients lying on a bed for dialysis treatments. Patients who are treated in dialysis chairs are not anticipated to have data on lying blood pressure measurements.<br>Note: Peritoneal dialysis patients will not have any data entered in this field and vital sign measurements are only captured in pre-dialysis fields.	mmHg	5	400
46	TXT_POST_TEMP	Num	Post-dialysis body temperature measurement.<br>Note: Peritoneal dialysis patients will not have any data entered in this field and vital sign measurements are only captured in pre-dialysis fields.	Celsius	15	50
47	TXT_POST_WEIGHT	Num	Post-dialysis weight measurement.<br>Note: Peritoneal dialysis patients will not have any data entered in this field and weight measurements are only captured in pre-dialysis field.	Kg	20	600
48	TXT_PRE_DBP	Num	Pre-dialysis sitting diastolic blood pressure (DBP) vital sign measurement. This includes data from patients treated in dialysis chairs.<br>Note: Peritoneal dialysis patients should have data entered in this field for vital sign measurements.	mmHg	5	400
49	TXT_PRE_POST_DILUTION	Char	Hemodiafiltration (HDF), whether HDF was performed with post-dilution, pre-dilution, or mixed- dilution technique for the infusion site of the substitution fluid. This field is only for HDF patients.			
50	TXT_PRE_PULSE	Num	Pre-dialysis pulse (i.e. heart rate) measurement. This is anticipated to be the measurement recorded while the patient was sitting or lying.<br>Note: Peritoneal dialysis patients should have data entered in this field for vital sign measurements.	beats/min	5	600
51	TXT_PRE_SBP	Num	Pre-dialysis sitting systolic blood pressure (SBP) vital sign measurement. This is anticipated to include data from patients treated in dialysis chairs.<br>Note: Peritoneal dialysis patients should have data entered in this field for vital sign measurements.	mmHg	5	400

52	TXT_PRE_TEMP	Num	Pre-dialysis body temperature measurement.<br>Note: Peritoneal dialysis patients should have data entered in this field for vital sign measurements.	Celsius	15	50
53	TXT_PRE_WEIGHT	Num	Pre-dialysis weight measurement.<br>Note: Peritoneal dialysis patients should data entered in this field for weight measurements.	Kg	20	600
54	TXT_QB	Num	Blood flow rate for hemodialysis or hemodiafiltration.	mL/min	0	2000
55	TXT_QD	Num	Dialysate flow rate for hemodialysis or hemodiafiltration.	mL/min	0	2000
56	TXT_START_TIME	Char	This is the local time the hemodialysis or hemodiafiltration treatment started, per the report from the dialysis machine or the charted time in the medical record.	hh:mm:ss	00:00:00	23:59:59
57	TXT_SUBSTITUTION_VOLUME	Num	Hemodiafiltration (HDF), total substitution/infusion volume. This is the total volume of substitution fluid the patient received during each HDF treatment. This field is only for HDF patients.	mL	0	100,000
58	TXT_TARGET_WEIGHT	Num	Target weight. This is goal weight to be achieved by the end of a hemodialysis or hemodiafiltration treatment on each day. Generally, this is determined by the nurse immediately before each treatment and based off the patients weight at each presentation and how much fluid needs to be removed to try to reach the estimated dry weight.	Kg	0	20
59	TXT_TIME	Num	Total treatment time. This is the duration of the hemodialysis or hemodiafiltration treatment the patient received on each day.	Minutes	0	1440
60	TXT_TIME_UF	Num	Total UF time. This is the total time that ultrafiltration (UF) was turned on during a hemodialysis or hemodiafiltration treatment.	Minutes	0	1440
61	TXT_TOTAL_FLUID	Num	Total fluid intake during dialysis + saline washback. This is all fluid the patient may have received including water drank during treatment (if recorded, saline administered during treatment, and the saline washback). If only saline administered during the treatment is recorded, this is entered in the field "TXT_TOTAL_SALINE"	mL	0	4000
62	TXT_TOTAL_SALINE	Num	Total saline administered during a hemodialysis or hemodiafiltration treatment. This is the total saline administered during the treatment (typically for intradialytic symptoms) and does not include the saline volume used for priming the blood lines.	mL	0	2000
63	TXT_UFV	Num	Ultrafiltration volume (UFV). This is the actual UFV that was achieved during hemodialysis (HD) or hemodiafiltration (HDF) for each patient on each treatment day. This is not intended to be the prescribe UFV volume, which may or may not be achieved during the delivery of HD or HDF. Please note that for HDF treatments, the UFV will be the total volume of fluid removed minus the substitution volume (basically, the fluid removed during dialysis from the patient's body not including infused substitution fluids used for HDF).	mL	-2000	20000
64	TXT_UF_PROFILE	Char	Ultrafiltration (UF) profile used during treatment. This is the UF profiling used during a hemodialysis or hemodiafiltration treatment. For Fresenius hemodialysis machines, there can be no UF profile set "none", or eight different profile settings.			
65	TXT_UKM_UREA	Num	Urea kinetic modeling (UKM) of the urea distribution volume for the patient. This is calculated by the hemodialysis or hemodiafiltration machine using the ultraviolet spectroscopy or ionic dialysance methods and represents an estimate of the patient's body volume that urea can be distributed throughout (considering a one compartment model). This is referred to as just "volume" on the adequacy (kt/v) screen for the Fresenius machine. This typically is about 30% to 80% of the patient body weight.	Liter	0	500
66	TXT_ACCESS_FLOW	Num	Flow monitoring of patients with an arteriovenous access.	mL/min	-4000	4000
67	TXT_IDWG_KG	Num	Interdialytic weight gain (IDWG). This is the weight gained between two hemodialysis or hemodiafiltration treatments.	Kg	-0.5	20
68	TXT_PD_DWELL_TIME_NIGHT	Num	Peritoneal dialysis: prescribed dwell time during the night. This is the time the peritoneal dialysate solution is prescribed to remain in the abdomen between exchanges during the night.	Minutes	1	1500
69	TXT_PD_EXCHG_NUM_NIGHT	Num	Peritoneal dialysis: number of exchanges of the dialysate during the night. For CCPD, this can be number of cycles if using cycler during the night.		0	20
70	TXT_PD_INFLOW_VOL_LAST	Num	Peritoneal dialysis: for patients using a cycler (CCPD), this is the volume of the inflow volume of the last fill before the daytime/long dwells during the time off the cycler until the next exchange or the reconnection to the cycler again (typically at night).	mL	1	10000
71	TXT_PD_INFLOW_VOL_NIGHT	Num	Peritoneal dialysis: prescribed inflow volume in the night. This is the volume of dialysate solution prescribed to go into abdomen for the night dwell(s).	mL	1	10000

QOL

Number	Variable	Variable Type	Description	Unit	Cleaning rules Min valid value	Cleaning rules Max valid value
1	GFME_ID	Num	**Primary key**: Randomly assigned patient identification number.			
2	DAYS_FROM_FDD	Char	Days between the first date of dialysis (FDD) for kidney failure (i.e. end stage kidney failure) and the date of the "event". In this table, the "event" is the KDQOL-36 survey being completed by a dialysis patient on that date (represented as days from FDD), as well as any associated data for the fields in this table. Please note that the reference KDQOL survey is the KDQOL-36 (not the KDQOL-SF).	Days		
3	QOL_Q1	Num	KDQOL-36 question 1: "In general, would you say your health is?"<br>For KDQOL-SF v1.3 survey, this is question 1<br>Raw data on responses are reported on a 5 point Likert scale.	Points	1	5
4	QOL_Q2	Num	KDQOL-36 question 2: "The following items are about activities you might do during a typical day. Does your health now limit you in these activities? If so, how much? ... Moderate activities, such as moving a table, pushing a vacuum cleaner, bowling, or playing golf"<br>For KDQOL-SF v1.3 survey, this is question 3b<br>Raw data on responses are reported on a 3 point Likert scale.	Points	1	3
5	QOL_Q3	Num	KDQOL-36 question 3: "The following items are about activities you might do during a typical day. Does your health now limit you in these activities? If so, how much? ... Climbing several flights of stairs"<br>For KDQOL-SF v1.3 survey, this is question 3d<br>Raw data on responses are reported on a 3 point Likert scale.	Points	1	3
6	QOL_Q4	Num	KDQOL-36 question 4: "During the past 4 weeks, have you had any of the following problems with your work or other regular daily activities as a result of your physical health? ... Accomplished less than you would like"<br>For KDQOL-SF v1.3 survey, this is question 4b<br>Raw data on responses are reported on a 2 point Likert scale.	Points	1	2
7	QOL_Q5	Num	KDQOL-36 question 5: "During the past 4 weeks, have you had any of the following problems with your work or other regular daily activities as a result of your physical health? ... Were limited in the kind of work or other activities."<br>For KDQOL-SF v1.3 survey, this is question 4c<br>Raw data on responses are reported on a 2 point Likert scale.	Points	1	2
8	QOL_Q6	Num	KDQOL-36 question 6: "During the past 4 weeks, have you had any of the following problems with your work or other regular daily activities as a result of any emotional problems (such as feeling depressed or anxious)? ... Accomplished less than you would like"<br>For KDQOL-SF v1.3 survey, this is question 5b<br>Raw data on responses are reported on a 2 point Likert scale.	Points	1	2
9	QOL_Q7	Num	KDQOL-36 question 7: "During the past 4 weeks, have you had any of the following problems with your work or other regular daily activities as a result of any emotional problems (such as feeling depressed or anxious)? ... Didn't do work or other activities as carefully as usual"<br>For KDQOL-SF v1.3 survey, this is question 5c<br>Raw data on responses are reported on a 2 point Likert scale.	Points	1	2
10	QOL_Q8	Num	KDQOL-36 question 8: "During the past 4 weeks, how much did pain interfere with your normal work (including both work outside the home and housework)?"<br>For KDQOL-SF v1.3 survey, this is question 8<br>Raw data on responses are reported on a 5 point Likert scale.	Points	1	5
11	QOL_Q9	Num	KDQOL-36 question 9: "How much of the time during the past 4 weeks ... Have you felt calm and peaceful?"<br>For KDQOL-SF v1.3 survey, this is question 9d<br>Raw data on responses are reported on a 6 point Likert scale.	Points	1	6
12	QOL_Q10	Num	KDQOL-36 question 10: "How much of the time during the past 4 weeks ... Did you have a lot of energy?"<br>For KDQOL-SF v1.3 survey, this is question 9e<br>Raw data on responses are reported on a 6 point Likert scale.	Points	1	6
13	QOL_Q11	Num	KDQOL-36 question 11: "How much of the time during the past 4 weeks ... Have you felt downhearted and blue?"<br>For KDQOL-SF v1.3 survey, this is question 9f<br>Raw data on responses are reported on a 6 point Likert scale.	Points	1	6
14	QOL_Q12	Num	KDQOL-36 question 12: "During the past 4 weeks, how much of the time has your physical health or emotional problems interfered with your social activities (like visiting with friends, relatives, etc.)?"<br>For KDQOL-SF v1.3 survey, this is question 10<br>Raw data on responses are reported on a 5 point Likert scale.	Points	1	5
